# Evaluation of thermal indices for their applicability in obstacle-resolving meteorology models

**DOI:** 10.1007/s00484-018-1591-6

**Published:** 2018-08-14

**Authors:** Jana Fischereit, K. Heinke Schlünzen

**Affiliations:** Meteorological Institute, CEN, Universität Hamburg, Hamburg, Germany

**Keywords:** Thermal environment, Outdoor, Numerical atmospheric modeling, Index evaluation, Microscale model

## Abstract

A thermally comfortable design of outdoor spaces favors social interaction and outdoor activities and thus contributes to the overall well-being of urban dwellers. To assess such a design, obstacle-resolving models (ORM) combined with thermal indices may be used. This paper reviews existing thermal indices to identify those suitable for thermal comfort assessment with ORMs. For the identification, 11 criteria and six index features are derived from literature analysis focusing on the characteristics of human environmental heat exchange, of outdoor urban environments, and of ORMs. An air temperature weighted world population distribution is calculated to derive the minimal air temperature range; a thermal index should cover to be applicable to 95% of the world population. The criteria are applied to 165 thermal indices by reviewing their original publications. Results show that only four thermal indices are suitable to be applied globally in their current form to various outdoor urban environments and also fulfill the requirements of ORMs. The evaluation of the index features shows that they differ with respect to the comprehensiveness of the thermophysiological model, the assessed human response, the treatment of clothing and activity, and the computational costs. Furthermore, they differ in their total application frequency in past ORM studies and in their application frequency for different climatic zones, as a systematic literature analysis of thermal comfort studies employing ORMs showed. By depicting the differences of the thermal indices, this paper provides guidance to select an appropriate thermal index for thermal comfort studies with ORMs.

**Electronic supplementary material:**

The online version of this article (10.1007/s00484-018-1591-6) contains supplementary material, which is available to authorized users.

## Introduction

Urban system models aim to describe the urban system consisting of individuals, society, morphology, and environmental stressors (von Szombathely et al. 2017) with its complex relations to model, for instance, health-related urban well-being. One important aspect of health-related urban well-being is the outdoor activity of urban dwellers. Not only does this positively affect individual health, but also the city as a whole can benefit from outdoor activities in physical, environmental, economical, and social ways (Chen and Ng 2012). To promote outdoor activities, an attractive design of public spaces is needed (Chen and Ng 2012). One aspect thereof is thermal comfort (Chen and Ng 2012). Thermally comfortable designs can be evaluated by thermal indices, since they summarize the effect of the thermal environment on the human body into one value (ASHRAE 2001; Parsons 2014). In total, at least 165 indices have been proposed (de Freitas and Grigorieva 2016). However, not all of them can be applied to assess *outdoor* thermal comfort because they neglect important parameters such as solar radiation or wind speed.

There are several reviews on the assessment of the thermal environment (Cheng et al. 2012; Djongyang et al. 2010; Goshayeshi et al. 2013; Walgama et al. 2006), including the reviews by Monteiro (2005) and Coccolo et al. (2016) that focus on the outdoor environment. However, no paper reviews the application of thermal indices in combination with obstacle-resolving numerical models of the atmosphere (from here on shorted ORM) such as ENVI-met (Bruse and Team 2015; Bruse and Fleer 1998) or MITRAS (Salim et al. 2018; Schlünzen et al. 2003), although those are increasingly used for urban planning (Goldberg et al. 2013) to assess different design strategies (e.g., implementation of vegetation and building orientation) as suggested by the German Engineering guideline (VDI 2008a).

ORMs are helpful to design public spaces in a comfortable way since different design scenarios can be evaluated for various meteorological conditions. Typically, ORM simulations have a domain size between 0.1 and 5 km with a spatial resolution of 0.1 to 100 m (Blocken 2015). Due to this high resolution, ORMs not only explicitly resolve buildings, but also the thermal conditions on temporal and spatial scales where people actually experience it.

Considering the large number of proposed indices and the characteristics of ORMs, this study addresses the overall question: *which thermal indices can be used globally in their current form to evaluate the outdoor thermal environment in ORM applications*? The term “in ORM applications” in this paper refers to an evaluation of the thermal situation simulated by the ORM by thermal indices, both online during the simulation and offline using model output. Since ORMs are applicable to design public spaces for urban residents, this study focusses on the typical urban resident and might not be directly applicable to outdoor workers or tourists.

This paper is structured as follows. In the section “Thermal indices and application demands,” the principles of thermal indices are introduced, and the characteristics of human environmental heat exchange of outdoor urban environments and of ORMs are summarized from a literature analysis. Based on those characteristics, criteria for suitable thermal indices are determined. The criteria are evaluated for the 165 indices included in the catalog by de Freitas and Grigorieva (2015) by reviewing their original literature and using the existing literature review by de Freitas and Grigorieva (2016) in the “Results” section. The “Discussion and conclusions” section summarizes and discusses the results and indicates prospects for further research.

## Thermal indices and application demands

This section describes the characteristics of human environmental heat exchange and the related concept of thermal indices (“Thermal indices and related definitions”), characteristics of outdoor urban environment (“Outdoor urban environments”), and characteristics of ORMs (“Obstacle-resolving atmospheric models (ORMs)”). Based on these characteristics, criteria for suitable thermal indices in ORM applications are derived along with additional features of suitable indices (“Evaluation procedure for suitable indices”).

### Thermal indices and related definitions

#### Human thermal environments and thermal indices

The human body exchanges heat with its surroundings by different processes: radiation, convection, evaporation, respiration, and, if a significant area is in direct contact with solid material, via conduction (Fiala and Havenith 2015). How much heat is exchanged via the different processes depends on four environmental variables, namely air temperature (*T*), humidity (*H*), wind speed (*v*), and long and shortwave radiation (*Q*^∗^, often summarized in the integrating variable mean radiant temperature, *T*_*mrt*_), and two human-related factors: activity and clothing. Activity controls the amount of heat produced by the body, and clothing insulation determines the resistance to heat exchange. All six factors together are referred to as the “six basic parameters” (Parsons 2014). The specific combination of the six basic parameters makes up the *human thermal environment* a person experiences. How a person feels in such a human thermal environment is defined as *thermal sensation*, e.g., hot, cold, or neutral. Thermal sensation cannot be expressed directly in physical or physiological terms as it is a psychological phenomenon. However, thermal sensations have been shown to correlate with environmental conditions and physiological responses of the human body (Parsons 2014).

A useful technique for the assessment of a thermal environment is the *thermal index*. The term “thermal index” is rarely defined in literature. Parsons (2014) defines an assessment of the thermal environment as an index, if it maps the factors that influence the human response to thermal environments to a single value that varies with the human response. This definition is applied in the present study.

#### Categories of thermal indices

Based on the measured human response, indices can be categorized into comfort or stress indices. *Thermal comfort* is defined as “that condition of mind which expresses satisfaction with the thermal environment” (ASHRAE 2001), whereas *thermal stress* quantifies the effect of the six basic parameters in terms of thermal strain experienced by the person (Parsons 2014).

Another possibility to categorize thermal indices has been proposed by MacPherson 1962, who discriminates direct, empirical, and rational indices. *Direct indices* are based on direct measurements of environmental variables, either by using integrated measurement devices, which model a human body, or by combining measured meteorological parameters using an algebraic weighted expression (MacPherson (1962), Eissing (1995)). In contrast, *empirical indices* are developed by exposing people to different environmental conditions (e.g., in a climate chamber) and measuring physiological parameters such as heart rate or rectal temperature. By means of multiple regression analysis, the different environmental conditions and possibly different clothing and activities are linked to the physiological reactions (MacPherson 1962). The third category, *rational indices*, formalizes the heat exchange mechanisms of the human body (“Human thermal environments and thermal indices”) to yield the heat balance equation (Eq. 1) of the human body (ASHRAE 2001; VDI 2008b):

1 $$ M+W+{Q}^{\ast }+{Q}_H+{Q}_L+{Q}_{SW}+{Q}_{Re}+S=0 $$

where *M* denotes metabolic heat; *W* mechanical work accomplished; *Q*^∗^ radiation budget; *Q*_*H*_, *Q*_*L*_, and *Q*_*SW*_ the turbulent flux of sensible heat, of latent heat by diffusion, and of latent heat by sweat evaporation; *Q*_*Re*_ the respiratory heat flux (sensible and latent); and *S* the rate of storage of heat. Individual heat fluxes are calculated from gradients between physiological variables such as skin temperature and environmental variables. The physiological state of a person results from regulation mechanisms in the body in response to the environmental conditions. The regulation mechanisms are simulated with different complexity in one-node, two-node, multi-node, and multi-element models (Cheng et al. 2012). Not only individual differences, such as gender and age (ASHRAE 2001; Rida et al. 2014), but also acclimatization (Froehle 2008) have been noted to influence the physiological thermoregulation. In addition to the thermoregulatory system of the body, people adapt to a stressful environment by changing their behavior (e.g., change in activity or exposure, Jendritzky and de Dear (2009)). Rational indices either refer to equilibrium conditions (*S* = 0), or to dynamic, transient conditions, or changing activities (*S* ≠ 0). Out of the three categories of indices, they have the most objective basis, since they are based on the first law of thermodynamics. However, empirical relationships are used to calculate the regulation mechanisms within the body (ASHRAE 2001).

Many indices apply the concept of a *standard* or *reference environment*. These thermal indices calculate the air temperature that would result in the “equivalent effect” for a person as the actual environment does, which consists of the six basic parameters (Parsons 2014). What is defined as “equivalent effect” depends on the individual index, e.g., some require the core temperature to be equal in both environments. These so-called equivalent temperatures have the same unit as air temperature and can therefore be understood by laypeople (Höppe 1999).

#### Assessment scales for indices

A thermal index value itself is not necessarily meaningful, since it depends on the assumptions of the underlying equations. It is not clear, for instance, whether an equilibrium temperature of 10 °C is desirable in terms of thermally optimal design, or a value of 25 °C is better. Therefore, an assessment scale is needed that maps individual index values into categories of similar and generally understood thermal sensations or thermal stresses.

Different types of assessment scales can be identified based on (1) strain reactions of the human body (e.g., Bröde et al. (2012)), (2) regression between accepted scales from climate chambers and index values (e.g., Matzarakis and Mayer (1996)), or (3) regression between thermal sensation votes (denoted TSV in the following) from surveys and index values (e.g., Watanabe et al. (2014)). The first two scales aim to predict the value of the thermal index for a clearly defined reference person who chooses a place freely without specific expectations and before it adapts to this particular thermal environment (Staiger et al. 2012). In contrast, scales derived from TSVs represent the thermal perception after adaptation and include cultural norms and expectations for people attending the place at a specific time without free choice (Staiger et al. 2012). Although TSVs are important to identify regional particularities, they are unsuitable for ORM applications, since they are valid only for the regional climatic context where they have been derived. The standardization initiative of thermal comfort studies (Johansson et al. 2014) may lead to a globally standardized database of TSVs. Those may be dense enough to be used in ORM applications; however, people who are deliberately avoiding the place due to uncomfortable environmental conditions are still not included in the TSVs, and thus TSVs may lead to skewed results (Staiger et al. 2012).

### Outdoor urban environments

#### Outdoor air temperature range

Outdoor thermal environments exhibit a much wider range of environmental parameters than controlled indoor environments (Jendritzky and de Dear 2009). To derive the air temperature range people are exposed to when being outdoors, two data sets have been combined. First, a global data set of observation-based monthly mean 2-m-air-temperature values (*T*) over land covering the period from 1986 to 2015 (Fan and van den Dool 2008), and second, a global data set for the population count (*P*) for the year 2000 (Center for International Earth Science Information Network - CIESIN - Columbia University et al. 2005). Both data sets have a resolution of 0.5^°^ × 0.5^°^. To estimate the air temperature range people are exposed to, an air temperature weighted population distribution is derived by calculating the population exposed (*PE*, Eq. 2) to a specific 5K-ΔT-range between 1986 and 2015:

2 $$ PE\ \left({T}_{\mathrm{min}}\right)=\frac{1}{N}\cdotp \left(\sum \limits_{m=1}^M\sum \limits_{i=1}^Nf\left({T}_{\mathrm{min}}\right)\cdotp {P}_i\right) $$

with

$$ f\left({T}_{\mathrm{min}}\right)=\left\{\begin{array}{l}1,{T}_{\mathrm{min}}\le T<{T}_{\mathrm{min}}+5K\\ {}0,\mathrm{else}\end{array}\right. $$

*M* is the number of months (*m*) between January 1986 and December 2015 (*M* = 360), *N* is the number of grid cells (index *i*), and *T*_min_ is varied between − 60 °C and 60 °C in 5 K-steps. For the air temperature data from 1986 to 2015 *T* lay between − 55.0 °C and 62.6 °C.

Due to slight differences in the land-sea-mask of the two data sets, about two million people (0.03% of the world population) could not be considered in the analysis. Most of them live on islands in the Pacific Ocean.

**Fig. 1:**
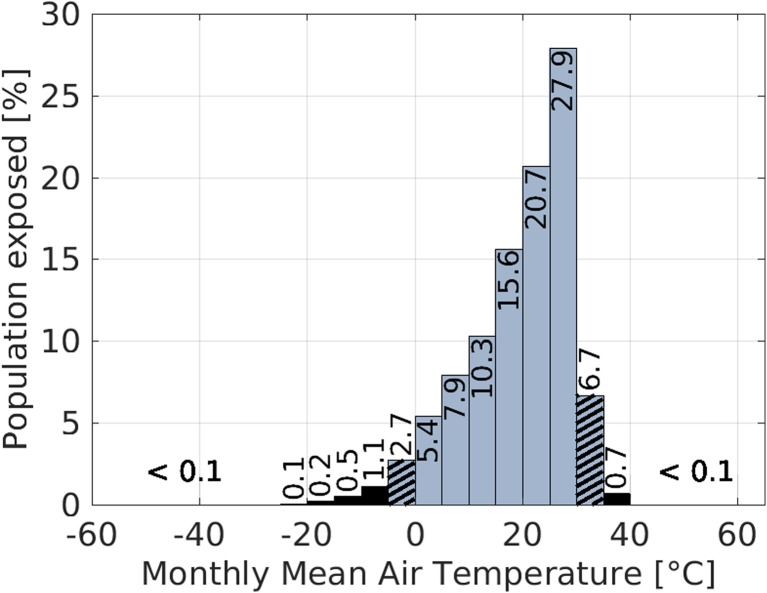
Percentage of the world population exposed to a specific 5 K monthly mean 2-m-air-temperature range. To each range below − 25 °C and above 40 °C less than 0.1% of the world’s population is exposed. Gray-colored bars indicate 95% of world population, hatched bars indicated the range containing the accumulated upper and lowermost 2.5% of the world population, and black-colored bars indicate the outer 5% of world population. Basic data have been taken from GHCN Gridded V2 data (Fan and van den Dool 2008) and the Gridded Population of the World dataset, Version 3 (see text)

Figure 1 shows *PE* for each Δ*T*-range. Only few people exposed to monthly mean air temperature values below – 25 °C or above 40 °C (less than 0.1% of the world’s population per range). Ninety-five percent of the world population lives in an air temperature range of − 5 °C to 35 °C. The hatched bars in Fig. 1 mark the two ranges enclosing 95% of the population.

#### Radiation fluxes and wind speed

A particular feature of outdoor environments is the presence of direct solar short-wave radiation fluxes. These include direct, diffuse, and reflected radiation fluxes. In an urban environment, long-wave radiation is not only emitted from the sky and the ground, but also from surrounding building walls. These walls, in turn, can shade areas and shield people from direct short-wave radiation.

The mean radiant temperature (*T*_*mrt*_), which is usually applied to express the effect of radiation (Kantor and Unger 2011), is the most variable parameter within an urban street canyon (Ali-Toudert and Mayer 2006; Chen et al. 2016; Jendritzky et al. 2007; Lee et al. 2016, 2014; Mayer et al. 2008). The second most variable parameter is the wind speed due to drag and advection effects. Radiation and wind are also those parameters that can best be modified for a thermally comfortable design (Barry and Blanken 2016) and affect thermal perception most (Moonen et al. 2012).

#### Urban residents’ activities and clothing behavior

Urban activities include standing, e.g., while smoking or talking, or walking, e.g., while shopping or commuting. Although activities vary for different types of urban spaces (Thorsson et al. 2007), in the current study, standing and walking are considered as typical urban activities as they reflect the typical behavior outside parks.

Clothing behavior in urban areas has been shown to vary seasonally (Havenith et al. 2011; Nikolopoulou et al. 2001) but within certain limits: Even in hot conditions, a minimum of 0.2 clo (1.0 clo is equivalent to a thermal resistance of clothing of 0.155 m^2^ K W^−1^, ASHRAE (2001)) has been observed, which corresponds for instance to short-sleeve shirt and short trousers (de Freitas 1987). Those limits might be due to cultural rules and norms (Knez et al. 2009). Urban clothing behaviors may differ significantly from clothing of beach tourists or workers wearing special protective clothes. Therefore, the indices selected in this study might not be applicable for those groups.

#### Persistence of outdoor environmental conditions

Today, many urban activities usually take place indoors: In most industrialized countries, people spend about 90% of their time inside buildings (Höppe 2002). Thus, the time spent outdoors is usually too short to achieve thermal equilibrium, especially as people tend to stray between different microclimates (Thorsson et al. 2007). Furthermore, the meteorological conditions are changing: A quasi-steady state, e.g., a state for which the thermal conditions of the body per time unit change only marginally, may be achieved for a certain microclimate within 2 h if the weather is constant, but not only the person might move, usually also the meteorological situation changes in that time (e.g., diurnal cycle). Therefore, an index considering dynamic conditions would be most suitable (“Categories of thermal indices”). However, such an index strongly depends on the thermal history of a person, e.g., exiting from a sauna or from an air-conditioned building. Therefore, to evaluate a certain design, simulations of an ensemble of people with different thermal histories would be required. However, getting that kind of information is difficult, and even then ensemble simulations are computationally intensive. Therefore, although dynamic indices are more realistic, steady state indices offer advantages for urban planning applications.

### Obstacle-resolving atmospheric models (ORMs)

#### Time scales

ORMs simulate thermal and dynamic atmospheric processes by numerically solving partial differential equations for conservation of energy, mass, and momentum. These so-called Navier-Stokes equations cannot be solved directly due to computational limitations (Blocken 2015). Hence, the equations are filtered and approximated. Nowadays, the time and space averaged so-called Reynold-Averaged-Navier-Stokes Equations (RANS) are used for simulating flows within urban areas (Blocken 2015). Those RANS models simulate the temporal mean flows in detail but with a typical time average of 10 to 20 min that mainly results from the parameterization of turbulent motion. The spatial resolution depends on the grid size used. RANS models are applied for studying urban areas (e.g., Ali-Toudert and Mayer 2006; Salim et al. 2015). For specific applications, quality guidelines are established (Franke et al. 2011; VDI 2017).

#### Input variables for thermal indices

By solving the RANS equations, ORMs simulate the temporal evolution and spatial distribution of several meteorological variables (e.g., air temperature and flow field, Bohnenstengel et al. 2004), which can then serve as an input for thermal indices. For the two human-related factors, clothing insulation and activity, standardized input tables have been established (e.g., ASHRAE 2001) which can be used to derive input values. In contrast, physiological input parameters such as heart rate or rectal temperature would require expert knowledge or a suitable thermophysiological model. Only if such a model exists for a particular index, it can be used in ORM applications.

#### Calculation of thermal indices in ORM applications

The calculation of thermal indices from ORM outputs requires either a set of equations or a suitable calculation program, since the manual estimation of index values from nomograms or tables is not feasible due to the high number of grid points in ORMs. In the past, several integrated measurement devices have been proposed for a convenient estimation of direct indices (“Categories of thermal indices”). Indices derived from those devices can be used in ORMs if either a methodology to model the device within the ORM or an equation fitted from standard meteorological parameters exists.

Indices can be calculated either on-line during the simulation or off-line using model output. From a physical point of view, an on-line calculation would only be necessary, if the heat released by a person impacts the surrounding atmosphere. Outdoors, a person’s impact on the thermal environment is small because the wind speed is large and the air is often well mixed. Indoors, the impact of persons on the air is commonly larger due to smaller exchange rates of air, and thus on-line coupling is attempted (e.g., Cropper et al. 2010). From a computational point of view, off-line calculation is favorable because the effect of a set of meteorological conditions can be estimated for different personal characteristics without the need to rerun the ORM. However, Buzan et al. (2015) showed for global simulations that infrequent model output can cause an underestimation of thermal stress experienced. To avoid this effect in ORMs, the output needs to be frequent enough to reflect the changing air temperature and wind conditions (e.g., about 20 min). The output might have to be even more frequent to capture changes in meteorology if the ORM is nested (Schlünzen et al. 2011).

#### Fields of application for ORMs

ORMs are applied for design and performance analysis of building components, pollutant dispersion, and wind and thermal comfort (Moonen et al. 2012). In terms of thermal comfort, various studies assess the impact of different urban features (vegetation, albedo, etc.) or building configurations on the human thermal environment (Jännicke et al. 2015; Lee et al. 2016; Moonen et al. 2012). To do so, it is essential that the index can evaluate the thermal environment at a specific location (not only relative to a different location) for a specific meteorological situation (no climate average values required as inputs). Frequently applied thermal indices allow for a comparison of thermally comfortable designs in different climatic zones.

### Evaluation procedure for suitable indices

From the characteristics described in Sections “Thermal indices and related definitions, Outdoor urban environments, and Obstacle-resolving atmospheric models (ORMs),” the following 11 selection criteria for determining indices suitable for ORM application are derived. A pre-condition for all indices selected is that they shall provide only one output value (“Human thermal environments and thermal indices”). The criteria cover input demands (C1, C2), calculation demands (C3–C9), and interpretation demands on the index (C10, C11). The numbering follows the order how an index would be applied in an ORM application:

1. The input of the index is retrievable from ORMs or from standardized tables (i.e., for activity and clothing; “Input variables for thermal indices”).
2. The index exploits meteorological input values on the same temporal scale as typical for output time scales of ORMs (“Fields of application for ORMs”).
3. The index is computable using a formula or a numerical model (“Calculation of thermal indices in ORM applications”).
4. The index assesses the local thermal environment at a specific location within an urban area (“Fields of application for ORMs”).
5. The index considers the influence of all six basic parameters (Temperature (*T*), humidity (*H*), wind speed (*v*), and radiation (*Q*^∗^), clothing and activity) in the calculation and includes both long-wave and short-wave radiative fluxes (“Human thermal environments and thermal indices” and “Radiation fluxes and wind speed”).
6. The index considers long-wave radiative fluxes from all directions (“Radiation fluxes and wind speed”).
7. The index considers the average air temperature range in which a large proportion of mankind lives (− 5 °C to 35 °C, “Outdoor air temperature range”).
8. The index considers typical clothing behavior and activities of urban residents (“Urban residents activities and clothing behavior”).
9. The index assesses thermal conditions for an exposure time of 10 min and more; instantaneous reactions should not be assessed (“Time scales”).
10. An assessment scale exists for the thermal index (“Assessment scales for indices”).
11. The assessment scale of the index is not derived from thermal sensation votes in a specific region (“Assessment scales for indices”).

The criteria are applied in the order given above (C1 to C11) to the 165 indices listed in the catalogue by de Freitas and Grigorieva (2015), which is the most comprehensive list of indices existing so far. If an index does not fulfill a specific criterion, subsequent criteria are not further assessed. To assess the criteria, the original literature of the indices has been reviewed. For 21 indices, the original literature could not be obtained and therefore secondary sources have been used. In our review of the original literature, differences have been found compared to the review by de Freitas and Grigorieva (2016). Those differences are described in Appendix B in ESM 1. For the analysis, indices are evaluated according to our review. For the three indices (“Perceived Temperature according to Linke,” “Physical saturation deficit,” and “Thermal Insulation of Clothing according to Aizenshtat”), the cited reference by de Freitas and Grigorieva (2016) did not contain such an index. Therefore, those indices had to be excluded from the analysis (Appendix B in ESM 1). For the index “Respiratory Heat Loss,” neither the original publication nor sufficient secondary literature could be obtained. Here, the review by de Freitas and Grigorieva (2016) was used, although it only allows an evaluation of some criteria (Appendix A in ESM 1). For criterion C7, the air temperature ranges given by de Freitas and Grigorieva (2016) are used for all indices.

After the 11 criteria are applied, for all remaining indices, six additional index features, also derived from “Thermal indices and related definitions” section to “Obstacle-resolving atmospheric models (ORMs)” section, are analyzed:

1. Unit of the thermal index (“Categories of thermal indices”)
2. Type of human response evaluated by the index (“Categories of thermal indices”)
3. Temporal resolution considered (“Persistence of outdoor environmental conditions”)
4. Implementation of index calculation in ORM applications (“Calculation of thermal indices in ORM applications”)
5. Available methods for the calculation of the index (“Calculation of thermal indices in ORM applications”)
6. Application frequency of the index in ORMs (“Fields of application for ORMs”)

The features F1 to F5 serve as information but do not lead to an exclusion of an index. The features are assessed by reviewing the original literature of the indices. For F6, a systematic literature review was performed using the databases “Scopus” (https://www.scopus.com/home.uri) and “Web of Science” (https://apps.webofknowledge.com) with the keywords “numerical model,” “thermal index,” “urban” including all fields in Scopus, and the topic in Web of Science on 15th November 2016. A total of 116 publications between 2000 and 2016 were obtained of which 106 were left after duplicates had been removed. By screening, 74 records were excluded because of at least one of the following reasons: (1) no ORM application, (2) study of a different spatial scale, (3) did not estimate a thermal index, or (4) were not published in a peer-reviewed journal. In total, 32 studies with different thermal indices remained to evaluate the application frequency (F6). The flow diagram and the 32 studies ordered by applied indices and by climatic zone are shown in Appendix C.

## Results

The assessment criteria derived in “Thermal indices and application demands” section are applied using the method described in “Evaluation procedure for suitable indices” section in order to identify suitable thermal indices for ORM applications.

### Application of criteria

From the 165 analyzed indices, two entries do not meet the definition of thermal indices used in this paper (“Human thermal environments and thermal indices”), since they provide more than one output (pre-condition for selected index): the Predicted effects of heat acclimatization (Givoni and Goldman 1973) and the Predicted Heat Strain (Malchaire et al. 2001). Therefore, they are not further analyzed.

**Fig. 2:**
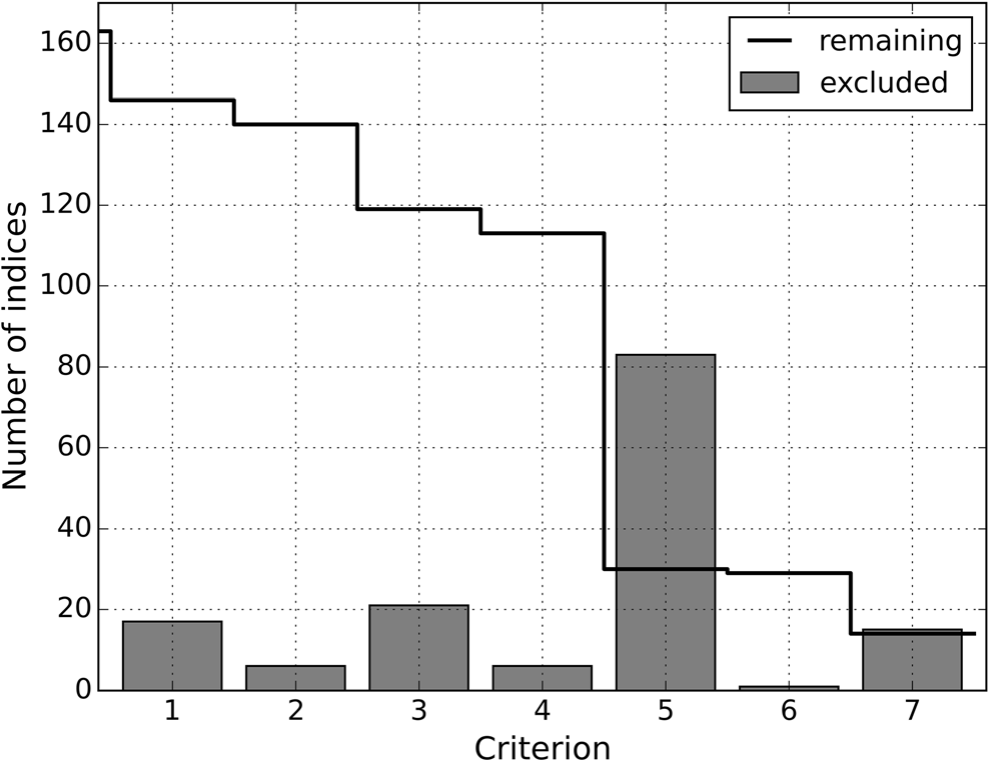
Number of indices excluded by criterion C1 to C7 (bars) and remaining number of indices (line). A detailed table of excluded indices is given in Appendix A in ESM 1

All indices excluded because of criterion C1 to C7 are shown in Appendix A in ESM 1 including their abbreviations, references, equations for their calculation as far as possible, as well as reasons for their exclusion. As noted before, the criteria are applied in the order given in “Evaluation procedure for suitable indices” section. If an index fails a criterion, subsequent criteria are not assessed. Figure 2 shows the number of indices excluded by C1 to C7 and the remaining number indices. Most indices do not consider all six basic parameters (C5). After C1 to C7 are applied, 13 indices remain. For those indices, the air temperature design ranges and restrictions for other meteorological variables are shown in Table 1.

**Table 1:** Air temperature design ranges (Δ*T*) of thermal indices meeting criteria C1 to C7. Ranges of wind speed in persons height (*v*) or 10 m (*v*_10_), relative humidity (*RH*), and mean radiant temperature (*T*_*mrt*_) are indicated as far as documented in the original publications. Air temperature ranges have been taken from de Freitas and Grigorieva (2016)

Δ*T* range [°C]	Index	Other ranges	Reference
− 25 ≤ *T* ≤ 35	Heat budget index (HEBIDEX) Skin temperature energy balance index (STEBIDEX)		de Freitas (1985); de Freitas (1986); de Freitas and Symon (1987)
− 40 ≤ *T* ≤ 40	Physiological strain (PhS), Subjective temperature index (STI)		Blazejczyk (2005b)
	Predicted mean vote—outdoors (PMV_o_)		Jendritzky and Nübler (1981)
	Physiological subjective temperature (PST)	*v* ≤ 22 ms^−1^	Blazejczyk et al. (2012); Blazejczyk and Matzarakis (2007)
− 40 ≤ *T* ≤ 50	Perceived temperature (PT_J_)		Jendritzky et al. (1979); Staiger et al. (2012)
− 50 ≤ *T* ≤ 50	Physiological equivalent temperature (PET)		Höppe (1999); (Mayer and Höppe 1987)
	Universal thermal climate index (regression, look-up table version; UTCI_app_)	0.5 ≤ *v*_10_ ≤ 30.3 ms^−1^, 5 ≤ *RH* ≤ 100%, − 30 ≤ *T*_*mrt*_ – *T* ≤ 70 °C	Bröde et al. (2012); Jendritzky et al. (2012)
− 90 ≤ *T* ≤ 37	Thermal balance (balance version, see Appendix A in ESM 1; ThBal_b_)		Rusanov (1981)
− 90 ≤ *T* ≤ 60	Outdoor thermal environment index (OTEI, ETVO)		Nagano and Horikoshi (2011)
	Universal thermal climate index (UTCI)		Bröde et al. (2012); Jendritzky et al. (2012)
	Standard effective temperature outdoors (OUT_SET*)		Pickup and de Dear (2000)

All 13 remaining indices (Table 1) clearly cover the air temperature range of − 5 °C to 35 °C (C7), where 95% of the world population lives (“Outdoor air temperature range”). For PST and UTCI_app_, additional restrictions concerning wind speed (both), relative humidity, and mean radiant temperature were found. Although no restrictions for the other indices were found, it is likely that their application range is also constrained, since the underlying parameterizations have been derived only for a limited number of conditions. C8 to C11 are applied to the indices in Table 1 (Table 2). Since all remaining indices are rational indices based on thermophysiological models for the human heat budget, they can be applied for every combination of clothing and activity. Therefore, no index is excluded due to criterion C8.

**Table 2:** Indices excluded from further analysis due to criterion (C). Full index names and references are shown in Table 1

C	Index	Reason
9	PhS	Evaluates reaction of body immediately after exposure to an environment (Blazejczyk 2005a; Blazejczyk 2005b). Thus, PhS evaluates time scales shorter than 10 min, which cannot be resolved with ORMs
9	STI	Same as PhS
10	OTEI	No assessment scale defined
10	ThBal_b_	No assessment scale defined. An assessment scale is defined for a regression version, but that does not include long-wave radiation (C5, Appendix A in ESM 1)
11	HEBIDEX	Assessment scale is derived from thermal sensation votes of beach tourists (de Freitas 1985)
11	STEBIDEX	Same as HEBIDEX
11	OUT_SET*	Contradicting assessment scales derived from thermal sensation votes for different locations by different authors (Tsitoura et al. (2014), Watanabe et al. (2014), and Spagnolo and de Dear (2003))

After assessing the indices with respect to C1 to C11, five indices, PMV_O_, PT_J_, PET, PST, and UTCI (and UTCI_app_) are found suitable for applications in ORMs. Since PT_J_ is an extension of PMV_O_ and improves the limited humidity-sensitivity in warm situations (Staiger et al. 2012), PMV_O_ is excluded from further analysis.

### Evaluation of index features

The indices PT_J_, PET, PST, UTCI_app_, and UTCI are analyzed with respect to their additional features (F1 to F6); the results are compared in Table 3. All remaining indices have a temperature unit (°C, F1). PT_J_ uses PMV to measure the equivalent effect and is therefore comfort-based (F2). Additionally, PT_J_ was linked to stress categories (Table 4, Staiger et al. (2012)). PET and UTCI also evaluate thermal stress (F2) since they use strain reactions to measure the equivalent effect in the reference environment and in the actual environment. PET is linked to the PMV scale via a linear regression (Matzarakis and Mayer 1996) and can therefore also be viewed as comfort-based (Blazejczyk et al. 2012). The validity of the regression method was questioned (e.g. Lee and Mayer 2016). Consequently for PET, other scales from TSVs have been derived for various climates (e.g., Lin and Matzarakis (2008); Holst and Mayer (2010); Kantor et al. (2012); Cohen et al. (2013)). However, these scales differ from the original scale (“Assessment scales for indices”) in terms of their implications. The categories of the UTCI assessment scale (Table 4) are derived from the occurrence of strain reactions such as the onset of shivering (Bröde et al. 2012). PST estimates thermal sensation (F2), but in contrast to the other indices is not an equilibrium temperature. Instead, PST is defined as the temperature established around the skin surface (under clothing) after 15–20 min of adaptation to maintain homeothermy. Therefore, the temporal resolution (F3) considered for PST is much more detailed than for UTCI (average over 2 h) and PT_J_ and PET, which estimate steady state conditions.

**Table 3:** Thermal indices for ORM applications fulfilling criteria C1 to C11. For entries related to features F1 to F6, the corresponding feature is given The following abbreviations are used: *A*_*Du*_ is body surface area, *BF* is body fat content, *e* is water vapor pressure, *e*_*a*_ is water vapor pressure under actual conditions (related to reference environments), *H* is a general measure for humidity with specification such as relative humidity (*RH*), *h* is height, *I*_*cl*_ is clothing insulation, *M* is metabolism, *m* is weight, *T* is air temperature, *T*_*cr*_ is core temperature, *T*_*mrt*_ is mean radiant temperature, *T*_*sk*_ is skin temperature, *v* is wind speed in person’s height, *v*_*w*_ is walking speed, *v*_10_ is wind speed in 10 m, and *W* is work metabolism. Superscripts have the following meaning: a Regression version of UTCI, b Look-up-table version of UTCI, and c full model version of UTCI. For index abbreviations see Table 1

Index	PT_J_	PET	PST	UTCI
Unit (F1)	°C	°C	°C	°C
Definition	Equilibrium temperature: same thermal perception (measured by PMV)	Equilibrium temperature: same *T*_*sk*_ and *T*_*cr*_	Temperature that is formed around skin surface (under clothing) after 15–20 min of adaptation to maintain homeothermy	Equilibrium temperature: same strain evaluated by same dynamic response of the physiological model
Reference conditions	*T*_*mrt*_ = *T*	*T*_*mrt*_ = *T*	Not applicable	*T*_*mrt*_ = *T*
	$$ H=\left\{\begin{array}{c}\ RH=50\%,\mathrm{warm}\\ {}\ e={e}_a,\kern1em \mathrm{else}\end{array}\right. $$	*e* = 12 hPa		$$ H=\left\{\begin{array}{c}e=20,\kern3.75em T>{29}^{{}^{\circ}}\mathrm{C}\\ {} RH=50\%,\kern0.5em \mathrm{else}\end{array}\right. $$
	*v* = 0.1 ms^−1^	*v* = 0.1 ms^−1^		*v*_10_ = 0.5 ms^−1^
Reference person	*M* = 135 Wm^−2^ (*v*_*w*_ = 4 kmh^−1^)	*M* ≈ 86 Wm^−2^ (W = 80 W)	*M* = 135 Wm^−2^ (*v*_*w*_ ≈ 4 kmh^−1^)	*M* ≈ 135 Wm^−2^ (*v*_*w*_ = 4 kmh^−1^)
	$$ {I}_{cl}=\left\{\begin{array}{c}1.75\ \mathrm{clo},\mathrm{winter}\\ {}f(T),\mathrm{else}\\ {}0.5\ \mathrm{clo},\mathrm{summer}\end{array}\right. $$	*I*_*cl*_ = 0.9 clo	$$ {I}_{cl}=\left\{\begin{array}{c}3\ \mathrm{clo},\mathrm{T}<-30\\ {}f(T),\mathrm{else}\kern0.75em \\ {}0.6\ \mathrm{clo},T>25\kern0.5em \end{array}\right. $$	*I*_*cl*_ = *f*(*T*) (Havenith et al. 2011)
	Male, 35 years	Male, 35 years		*BF* = 14%
	*m* = 75 kg,	*m* = 75 kg		*m* = 73.4 kg
	*A*_*Du*_ = 1.9 m^2^	*A*_*Du*_ = 1.9 m^2^		*A*_*Du*_ = 1.85 m^2^
	*h* = 1.75 m	*h* = 1.75 m		
Measure of assessment scale (F2)	Thermal perception; thermophysiological stress, directly linked to PMV-scale	Thermophysiological stress, related to PMV-scale	Thermal sensation	Thermal stress
Temporal resolution (F3)	Steady state	Steady state	After 15 to 20 min exposure	Average over 2 h
Thermophysiological model (related to F4)	Klima-Michel-Model (KMM), parameterizations derived from a two-node model (Gagge et al. 1986)	Munich energy balance model for individuals (MEMI), two-node	Man-ENvironment heat EXchange model (MENEX), one-node	UTCI-Fiala Model, multi-element
Coupling (F4)	On-line/off-line	Off-line	On-line/off-line	On-line^a^, off-line^b,c^
Code availability (F5)	VDI (2008b)	VDI (2008b)	No	Version^a,b^ via ISB Commission 6 (2012)
Software (examples, F5)	Free software RayMan (Matzarakis and Fröhlich 2009; Matzarakis et al. 2007; Matzarakis et al. 2010), sub-module BioMet of the commercial version of ENVI-met (Bruse and Team 2015)	Free software package RayMan (Matzarakis and Fröhlich 2009; Matzarakis et al. 2007; Matzarakis et al. 2010), sub-module Bio-met of the commercial version of ENVI-met (Bruse and Team 2015)	Free software BioKlima (Blazejczyk 2010)	Free software package RayMan (Matzarakis and Fröhlich 2009; Matzarakis et al. 2007; Matzarakis et al. 2010) and BioKlima (Blazejczyk 2010), sub-module Bio-met of the commercial version of ENVI-met (Bruse and Team 2015)
Assessment scale (F6)	See Table 4			
Ranges of meteorological inputs	See Table 1			
Reference	Jendritzky et al. (1990); Staiger et al. 2012	Mayer and Höppe (1987); Höppe (1999)	Blazejczyk and Matzarakis (2007); Blazejczyk et al. (2012)	Bröde et al. (2012); Jendritzky et al. (2012)

**Table 4:** Assessment scales of thermal indices suitable for ORM applications based on criteria C1 to C11. For index abbreviations see Table 1. Physiological stress categories refer to PT_J_, PET, and UTCI but not to PST

Thermal sensation	PST [°C]	PT_J_ [°C]	PET[°C]	UTCI[°C]	Physiological Stress
+ 5 sweltering	≥ 54				
+ 4 very hot	44 to 54	≥ 38	≥ 41	> 46	Extreme heat stress
				38 to 46	Very strong heat stress
+ 3 hot	34 to 44	32 to 38	35 to 41	32 to 38	Strong heat stress
+ 2 warm	24 to 34	26 to 32	29 to 35	26 to 32	Moderate heat stress
+ 1 slightly warm		20 to 26	23 to 29		Slight heat stress
0 neutral (comfortable)	14 to 24	0 to 20	18 to 23	9 to 26	No thermal stress
− 1 slightly cool		− 13 to 0	13 to 18	0 to 9	Slight cold stress
− 2 cool	4 to 14	− 26 to − 13	8 to 13	− 13 to 0	Moderate cold stress
− 3 cold	− 16 to 4	− 39 to − 26	4 to 8	− 27 to − 13	Strong cold stress
− 4 very cold	− 36 to − 16	≤− 39	≤ 4	− 40 to − 27	Very strong cold stress
− 5 frosty	≤− 3			<− 40	Extreme cold stress

Whether an index is capable to be applied on-line (F4) depends primarily on the computational cost required for the calculation. The computational cost can be estimated from the evaluated temporal state (e.g., more calculations are needed to reach thermal equilibrium) and the complexity of the thermophysiological model (e.g., more complex multi-elements models require more calculations). Temporal state, the thermophysiological models, and the derived on-line or off-line application type are shown in Table 3. The thermophysiological model of PST (MENEX) is a one-node model. Due to the nature of a one-node model, PST cannot account for thermophysiological regulation processes within the body, e.g., heat exchange between different body parts. To consider these processes, at least two nodes are necessary (“Categories of thermal indices”) as considered in thermophysiological models of PET and PT_J_. Out of the thermophysiological models of four suitable indices, the UTCI-Fiala model is the most sophisticated. Due to its multi-element structure, it predicts the state of individual body parts, although the UTCI index itself currently represents an entire body value.

The software (F5) to calculate the indices is indicated in Table 3. The source code is only publically available for UTCI_app_. For PET and PT_J_, source code is available from VDI (2008b).

PT_J_, PET, PST, and UTCI not only differ with respect to the index features but also regarding the treatment of clothing and activity (criterion C8). PET uses a fixed clothing insulation of 0.9 clo for the definition of the assessment scale (Table 3). Hence, it is a purely climatic index independent of individual behavior (Höppe 1999). However, other clothing values may be used in MEMI, although the assessment scale is technically applicable only for 0.9 clo. The three other indices account for a behavioral adjustment of clothing. In the calculation of UTCI, a full clothing model is incorporated (Havenith et al. 2011), which considers typical clothing behavior of urban residents, derived from studies in Europe and Russia. By considering adjustable clothing, behavioral adaptation (“Categories of thermal indices”) is accounted for. For the wide range of atmospheric conditions experienced outdoors, fixed clothing is unlikely to represent the clothing behavior of the population during all seasons. However, to be able to compare thermal climates at two locations, fixed clothing may be preferred. PET considers a very light activity (standing still), which represents the lowest expected outdoor body heat production under normal circumstances. The three other indices consider walking at 4 km h^−1^. The UTCI index is currently further developed to include other clothing and activity levels (Bröde et al. 2016).

The indicated assumptions and limitations of individual indices must be kept in mind by the user when applying these indices. Despite the differences between the indices, they have been shown to be strongly correlated (e.g., Blazejczyk et al. (2012); Staiger et al. (2012); Park et al. (2014); Matzarakis et al. (2014); Fröhlich and Matzarakis (2015)). The correlations, however, were found to be regime dependent (Staiger et al. (2012); Fröhlich and Matzarakis (2015)) because of the sensitivity of the indices to specific meteorological parameters and the different clothing models.

**Fig. 3:**
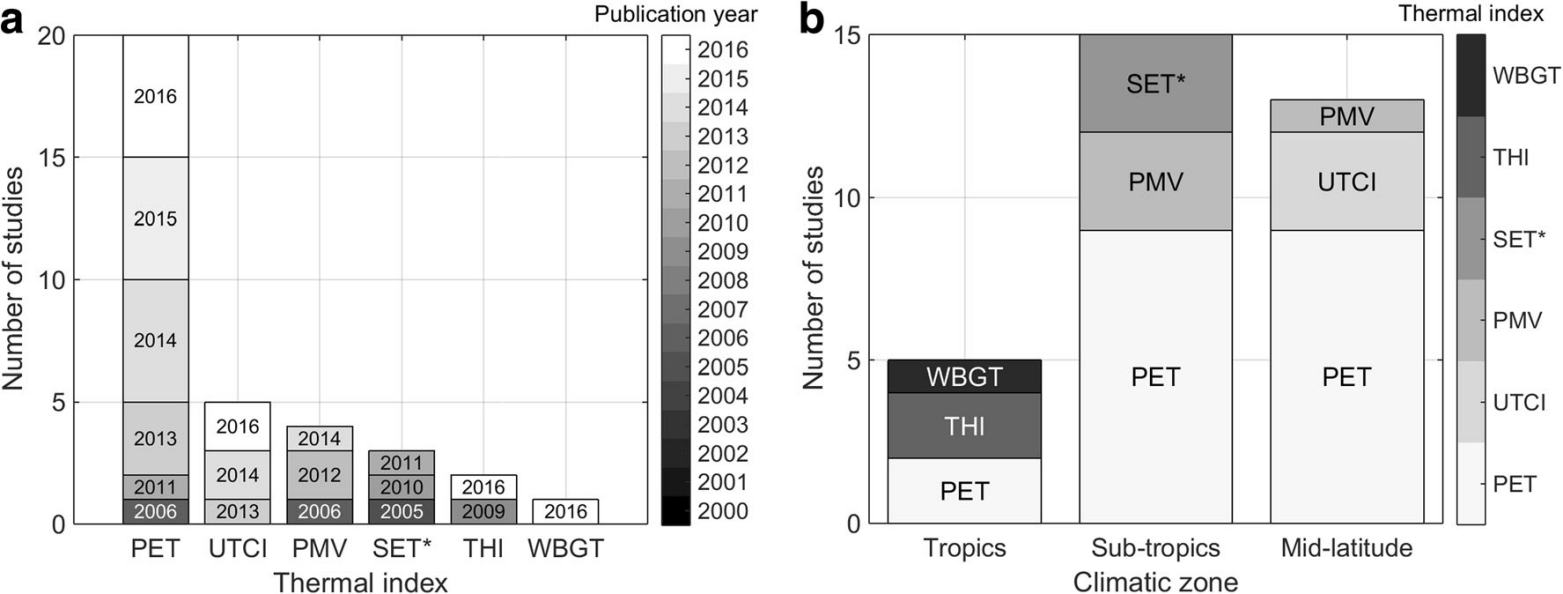
Number of ORM applications using the different indices published in different years (gray-colored) (a) and per climate zone (indices gray-colored) (b). Appendix C in ESM 1 summarizes the studies included in the analysis derived from the method in “Evaluation procedure for suitable indices” section. For abbreviations of indices see Appendix A in ESM 1 and Table 1. Note that some studies applied several indices and that PMV and SET* are used here to summarize studies that apply these indices in their original or derived form

For the evaluation of F6, a systematic literature review was conducted as described in “Evaluation procedure for suitable indices” section. Figure 3 shows the results of the 32 identified studies (references are given in Appendix C in ESM 1). PET is the most widely applied index in ORM applications (Fig. 3a). It remained popular even after the development of UTCI in 2012. Similar results were obtained by Coccolo et al. (2016), who did not focus on the microscale. PET has been applied in all three climatic zones, whereas most other indices have been applied only in some zones (Fig. 3b). Most studies have been conducted for the subtropics, followed by temperate climate and the tropics. No study for polar climate was found in the systematic review.

This statistical analysis shows that of the selected indices in this study, only PET has been applied in different climatic zones. PET is also the most frequently applied index. Therefore, PET is most suitable for comparing simulation results for different cities around the globe. No studies were found to apply PST or PT_J_, although with PMV, a precursor of PT_J_ was applied. The two indices without a rational basis (THI, WBGT; Appendix A) are least frequently applied; Morakinyo et al. (2016) use them in addition to PET.

## Discussion and conclusions

This paper identifies thermal indices suitable for ORM applications in urban environments for evaluating thermally comfortable designs. For the identification, 11 criteria have been derived based on the characteristics of human-environmental heat exchange (“Thermal indices and related definitions”), of outdoor urban environments (“Outdoor urban environments”), and of ORMs (“Obstacle-resolving atmospheric models (ORMs)”). The criteria were developed to identify indices that can be applied in ORMs in their current version globally without too much consideration by the users. The criteria have been evaluated for 165 indices listed by de Freitas and Grigorieva (2016) by inspecting the original literature. In total, four indices (PET, PT_J_, PST, and UTCI) with different characteristics (“Evaluation of index features”) have been found to fulfill all criteria.

Indices not selected in this analysis are not “bad” but not targeted for the intended type of application. Indices developed in form of nomograms, for instance, can be transformed to a program but that would require extra work from the user (C3). This may change, however, if the index is further developed. The indices selected in this study were targeted for the average urban resident with typical urban clothing and activity. To assess the thermal environment of beach tourists or workers wearing special protective clothes, different indices may be needed than those selected in this study. Additionally, only those indices were selected that can be applied to an air temperature range of − 5 °C to 35 °C. This range was determined to cover the climatic air temperature range 95% of the world population experiences. If only warm conditions shall be thermally assessed, all indices discarded by C7 in Appendix A in ESM 1 might be usable. For those indices criteria, C8 to C11 should be evaluated before use. Besides the temperature design range also the ranges for humidity, wind, and radiation to which the world population is exposed to should be evaluated. However, only for very few indices, the applicable design ranges for those parameters are given in the original literature. Therefore, this has not been attempted here. For future index developments, the design range of all input parameters should be clearly defined.

In urban planning, a design should result in thermal comfort for the average population. The thermal indices selected in this study assess the average population by considering a reference person (Table 3). As a result, however, the individual perception of a specific environment may differ from the assessment calculated by the index. Individual perception depends on the thermal history (“Persistence of outdoor environmental conditions”), the expectations of an individual and the interaction with other stressors such as noise or odors (Coccolo et al. 2016). Although important, this multitude of factors currently cannot be taken into account when designing thermal comfortable spaces for the general population. However, with increasing computational power and increased knowledge on human behavior, new methods for thermal environmental assessment in ORM applications in the context of urban planning may be established. Computational power may favor application of turbulence resolving ORMs, for which a suitable index should be able to consider the unsteadiness of the flow (Fanger et al. 1988). Furthermore, ensemble simulations for individuals with different personal characteristics and thermal histories could be used to evaluate the environment dynamically, as recommended for outdoor applications by Höppe (2002) and Coccolo et al. (2016). First steps in this direction have been taken by Bruse (2007). In the modeling framework of urban system models (von Szombathely et al. 2017), all those interactions could be combined to model health-related urban well-being. By extending studies such as by Hoffmann et al. (2018) to realistic cases, ORMs along with the found suitable thermal indices can make up one component in a suite of different multi-sectorial models to model the entire urban system.

## Electronic supplementary material

ESM 1 (PDF 839 kb)

## References

[CR1] Ali-Toudert F, Mayer H (2006). Numerical study on the effects of aspect ratio and orientation of an urban street canyon on outdoor thermal comfort in hot and dry climate. Build Environ.

[CR2] ASHRAE (2001). ASHRAE handbook: fundamentals.

[CR3] Barry RG, Blanken PD (2016). Microclimate and local climate.

[CR4] Blazejczyk K (2005a) MENEX_2005 - the updated version of man-environment heat exchange model. http://www.igipz.pan.pl/tl_files/igipz/ZGiK/opracowania/indywidualne/blazejczyk/MENEX_2005.pdf, last accessed 2017–06-13

[CR5] Blazejczyk K (2005b) New indices to assess thermal risks outdoors. In: environmental ergonomics XI, Proceedings of 11th international conference, Ystat, Sweden. Holmer, I and Kuklane, I. and Gao, Ch., pp 222–225

[CR6] Blazejczyk K (2010) BioKlima - universal tool for bioclimatic and thermophysiological studies. http://www.igipz.pan.pl/Bioklima-zgik.html. Accessed 27.10.2016

[CR7] Blazejczyk K, Matzarakis A (2007). Assessment of bioclimatic differentiation of Poland based on the human heat balance. Geogr Pol.

[CR8] Blazejczyk K, Epstein Y, Jendritzky G, Staiger H, Tinz B (2012). Comparison of UTCI to selected thermal indices. Int J Biometeorol.

[CR9] Blocken B (2015). Computational fluid dynamics for urban physics: importance, scales, possibilities, limitations and ten tips and tricks towards accurate and reliable simulations. Build Environ.

[CR10] Bohnenstengel SI, Schlünzen KH, Grawe D (2004). Influence of thermal effects on street Canyon Circulations. Meteorol Z.

[CR11] Bröde P (2012). Deriving the operational procedure for the universal thermal climate index (UTCI). Int J Biometeorol.

[CR12] Bröde P, Kampmann B, Fiala D (2016) Extending the Universal Theraml Climate Index UTCI towards varying activity levels and exposure times. Paper presented at the 9th Windsor conference: making comfort relevant, Cumberland lodge, Windsor, UK

[CR13] Bruse M (2007) Simulating human thermal comfort and resulting usage patterns of urban open spaces with a multi-agent system. In: Wittkopf S, Tan BK (eds) Proceedings of the 24th international conference on passive and low energy architecture PLEA, Singapore

[CR14] Bruse and Team (2015) ENVI-met BioMet. http://www.model.envi-met.com/hg2e/doku.php?id=apps:biomet. Accessed 27.10.2016

[CR15] Bruse M, Fleer H (1998). Simulating surface–plant–air interactions inside urban environments with a three dimensional numerical model. Environ Model Softw.

[CR16] Buzan JR, Oleson K, Huber M (2015). Implementation and comparison of a suite of heat stress metrics within the community land model version 4.5. Geosci Model Dev.

[CR17] Center for International Earth Science Information Network - CIESIN - Columbia University, United Nations Food + Agriculture Programme - FAO, Centro Internacional de Agricultura Tropical - CIAT (2005) Gridded population of the world, version 3 (GPWv3): population count grid. NASA Socioeconomic Data and Applications Center (SEDAC), Palisades, NY, 10.7927/H4639MPP, Accessed 2017–05-22

[CR18] Chen L, Ng E (2012). Outdoor thermal comfort and outdoor activities: a review of research in the past decade. Cities.

[CR19] Chen L, Yu B, Yang F, Mayer H (2016). Intra-urban differences of mean radiant temperature in different urban settings in Shanghai and implications for heat stress under heat waves: a GIS-based approach. Energy and Buildings.

[CR20] Cheng Y, Niu J, Gao N (2012). Thermal comfort models: a review and numerical investigation. Build Environ.

[CR21] CIESIN (2005) Gridded population of the world, version 3 (GPWv3) data collection, geospatial data presentation form: raster digital data, map. Socioeconomic Data and Applications Center (SEDAC), Columbia University. Available at http://sedac.ciesin.columbia.edu/gpw/index.jsp, Palisades, NY

[CR22] Coccolo S, Kämpf J, Scartezziani J-L, Pearlmutter D (2016). Outdoor human comfort and thermal stress: a comprehensive review on models and standards. Urban Clim.

[CR23] Cohen P, Potchter O, Matzarakis A (2013). Human thermal perception of coastal Mediterranean outdoor urban environments. Appl Geogr.

[CR24] Cropper PC, Yang T, Cook M, Fiala D, Yousaf R (2010). Coupling a model of human thermoregulation with computational fluid dynamics for predicting human–environment interaction. J Build Perform Simul.

[CR25] de Freitas C (1985). Assessment of human bioclimate based on thermal response. Int J Biometeorol.

[CR26] de Freitas CR (1986) Human thermal climates of New Zealand. New Zealand Meteorological Service, Misk Publ, 190, Wellington

[CR27] de Freitas CR (1987). Bioclimates of heat and cold stress in New Zealand. Weather and Climate.

[CR28] de Freitas C, Grigorieva E (2015). A comprehensive catalogue and classification of human thermal climate indices. Int J Biometeorol.

[CR29] de Freitas C, Grigorieva E (2016) A comparison and appraisal of a comprehensive range of human thermal climate indices. Int J Biometeorol. 10.1007/s00484-016-1228-6 10.1007/s00484-016-1228-6 27568190

[CR30] de Freitas CR, Symon LV (1987). A bioclimatic index of human survival times in the Antarctic. Polar Rec.

[CR31] Djongyang N, Tchinda R, Njomo D (2010). Thermal comfort: a review paper. Renew Sust Energ Rev.

[CR32] Eissing G (1995). Climate assessment indices. Ergonomics.

[CR33] Fan Y, van den Dool H (2008). A global monthly land surface air temperature analysis for 1948–present. J Geophys Res.

[CR34] Fanger PO, Melikov AK, Hanzawa H, Ring J (1988). Air turbulence and sensation of draught. Energ Buildings.

[CR35] Fiala D, Havenith G (2015) Modelling human heat transfer and temperature regulation. In: Gefen A, Epstein Y (eds) The mechanobiology and mechanophysiology of military-related injuries. Studies in Mechanobiology, Tissue Engineering and Biomaterials, vol 19. pp 265–302

[CR36] Franke J, Hellsten A, Schlünzen KH, Carissimo B (2011). The COST 732 best practice guideline for CFD simulation of flows in the urban environment - a summary. Int J Environ Pollut.

[CR37] Froehle A (2008). Climate variables as predictors of basal metabolic rate: new equations. Am J Hum Biol.

[CR38] Fröhlich D, Matzarakis A (2015). A quantiative sensitivity analysis on the behaviour of common thermal indices under hot and windy conditions in Doha Qatar. Theor Appl Climatol.

[CR39] Gagge AP, Fobelets AP, Berglund LG (1986). A standard predictive index of human response to the thermal environment. ASHRAE Trans.

[CR40] Givoni B, Goldman RF (1973). Predicting heart rate response to work, environment, and clothing. J Appl Physiol.

[CR41] Goldberg V, Kurbjuhn C, Bernhofer C (2013). How relevant is urban planning for the thermal comfort of pedestrians? Numerical case studies in two districts of the city of Dresden (Saxony/Germany). Meteorol Z.

[CR42] Goshayeshi D, Shahidan MF, Khafi F, Ehtesham E (2013). A review of researches about human thermal comfort in semi-outdoor spaces. Eur Online J Nat Soc Sci.

[CR43] Havenith G (2011). The UTCI-clothing model. Int J Biometeorol.

[CR44] Hoffmann P, Fischereit J, Heitmann S, Schlünzen K, Gasser I (2018). Modeling exposure to heat stress with a simple urban model. Urban Sci.

[CR45] Holst J, Mayer H (2010). Urban human-biometeorology: investigations in Freiburg (Germany) on human thermal comfort. Urban Climate News.

[CR46] Höppe P (1999). The physiological equivalent temperature – a universal index for the biometeorological assessment of the thermal environment. Int J Biometeorol.

[CR47] Höppe P (2002). Different aspects of assessing indoor and outdoor thermal comfort. Energ Buildings.

[CR48] ISB Comission 6 (2012) UTCI universal thermal climate index - documents. http://www.utci.org/utci_doku.php. Accessed 27.10.2016

[CR49] Jännicke B, Meier F, Hoelscher M-T, Scherer D (2015). Evaluating the effects of facade greening on human bioclimate in a complex urban environment. Adv Meteorol.

[CR50] Jendritzky G, de Dear R (2009). Adaptation and thermal environment.

[CR51] Jendritzky G, Nübler W (1981). A model analysing the urban thermal environment in physiologically significant terms. AMGBB.

[CR52] Jendritzky G, Sönning W, Swantes HJ (1979). Ein objektives Bewertungsverfahren zur Beschreibung des thermischen Milieus in der Stadt- und Landschaftsplanung ("Klima-Michel-Modell").

[CR53] Jendritzky G, Menz G, Schmidt-Kessen W, Schirmer H (1990). Methodik zur raumbezogenen Bewertung der thermischen Komponente des Bioklima des Menschen (Fortgeschriebenes Klima-Michel-Modell).

[CR54] Jendritzky G, Fiala D, Havenith G, Koppe C, Laschewski G, Staiger H, Tinz B (2007). Thermische Umweltbedingungen promet. Biometeorologie des Menschen.

[CR55] Jendritzky G, de Dear R, Havenith G (2012). UTCI—why another thermal index?. Int J Biometeorol.

[CR56] Johansson E, Thorsson S, Emmanuel R, Krüger E (2014). Instruments and methods in outdoor thermal comfort studies – the need for standardization. Urban Clim.

[CR57] Kantor N, Unger J (2011). The most problematic variable in the course of human-biometeorological comfort assessment - the mean radiant temperature. Cent Eur J Geosci.

[CR58] Kantor N, Unger J, Gulyas A (2012). Subjective estimations of thermal environment in recreational urban spaces—part 2: international comparison. Int J Biometeorol.

[CR59] Knez I, Thorsson S, Eliasson I, Lindberg F (2009). Psychological mechanisms in outdoor place and weather assessment: towards a conceptual model. Int J Biometeorol.

[CR60] Lee H, Mayer H (2016). Validation of the mean radiant temperature simulated by the RayMan software in urban environments. Int J Biometeorol.

[CR61] Lee H, Mayer H, Schindler D (2014). Importance of 3-D radiant flux densities for outdoor human thermal comfort on clear-sky summer days in Freiburg, Southwest Germany. Meteorol Z.

[CR62] Lee H, Mayer H, Chen L (2016). Contribution of trees and grasslands to the mitigation of human heat stress in a residential district of Freiburg, Southwest Germany. Landsc Urban Plan.

[CR63] Lin T-P, Matzarakis A (2008). Tourism climate and thermal comfort in Sun Moon Lake, Taiwan. Int J Biometeorol.

[CR64] MacPherson RK (1962). The assessment of the thermal environment. A review. Br J Ind Med.

[CR65] Malchaire J (2001). Development and validation of the predicted heat strain model. Ann Occup Hyg.

[CR66] Matzarakis A, Fröhlich D (2009) RayMan. http://www.urbanclimate.net/rayman/. Accessed 27.10.2016

[CR67] Matzarakis A, Mayer H (1996). Another kind of environmental stress: thermal stress WHO Colloborating Centre for Air Quality Management and Air Pollution Control. Newsletter.

[CR68] Matzarakis A, Rutz F, Mayer H (2007). Modelling radiation fluxes in simple and complex environments—application of the RayMan model. Int J Biometeorol.

[CR69] Matzarakis A, Rutz F, Mayer H (2010). Modelling radiation uxes in simple and complex environments - application of the rayman model. Int J Biometeorol.

[CR70] Matzarakis A, Muthers S, Rutz F (2014). Application and comparison of UTCI and PET in temperate climate conditions. Finisterra - Revista Portuguesa de Geografia.

[CR71] Mayer H, Höppe P (1987). Thermal comfort of man in different urban environments. Theor Appl Climatol.

[CR72] Mayer HHJ, Dostal P, Imbery F, Schindler D (2008). Human thermal comfort in summer within an urban street canyon in Central Europe. Meteorol Z.

[CR73] Monteiro LM (2005) review of numerical modelling of outdoor thermal comfort. In: The 2005 world sustainable building conference, Tokyo, pp 2252–2259

[CR74] Moonen P, Defraeye T, Dorer V, Blocken B, Carmeliet J (2012). Urban physics: effect of the micro-climate on comfort, health and energy demand. Frontiers of Architectural Research.

[CR75] Morakinyo TE, Dahanayake KWDKC, Adegun OB, Balogun AA (2016). Modelling the effect of tree-shading on summer indoor and outdoor thermal condition of two similar buildings in a Nigerian university. Energ Buildings.

[CR76] Nagano K, Horikoshi T (2011). Development of outdoor thermal index indicating universal and separate effects on human thermal comfort. Int J Biometeorol.

[CR77] Nikolopoulou M, Baker N, Steemers K (2001). Thermal comfort in outdoor urban spaces: understanding the human parameter. Sol Energy.

[CR78] Park S, Tuller S, Jo M (2014). Application of universal thermal climate index (UTCI) for microclimatic analysis in urban thermal environments. Landsc Urban Plan.

[CR79] Parsons KC (2014). Human thermal environments: the effects of hot, moderate, and cold environments on human health, comfort, and performance.

[CR80] Pickup J, de Dear RJ (2000) An outdoor thermal comfort index (OUT_SET*) - part I - the model and its assumptions. In: de Dear RJ, Kalma JD, Oke TR, Auliciems A (eds) Biometeorology and urban climatology at the turn of the millennium: selected papers from the conference ICB-ICUC'99

[CR81] Rida M, Ghaddar N, Ghali K, Hoballah J (2014). Elderly bioheat modeling: changes in physiology, thermoregulation, and blood flow circulation. IntJ Biometeorol.

[CR82] Rusanov V (1981). Complex meteorological indices and methods of climate assessment in medical purposes.

[CR83] Salim MMH, Schlünzen KH, Grawe D (2015). Including trees in the numerical simulations of the wind flow in urban areas: should we care?. J Wind Eng Ind Aerodyn.

[CR84] Salim MH, Schlünzen KH, Grawe D, Boettcher M, Gierisch AMU, Fock BH (2018). The microscale obstacle resolving meteorological model MITRAS: model theory. Geosci Model Dev Discuss.

[CR85] Schlünzen KH (2003). Flow and transport in the obstacle layer: first results of the micro-scale model MITRAS. J Atmos Chem.

[CR86] Schlünzen KH, Grawe D, Bohnenstengel SI, Schlüter I, Koppmann R (2011) Joint modelling of obstacle induced and mesoscale changes - current limitations and challenges. J Wind Eng Ind Aerodyn:217–225. 10.1016/j.jweia.2011.01.009

[CR87] Spagnolo J, de Dear R (2003). A field study of thermal comfort in outdoor and semi-outdoor environments in subtropical Sydney Australia. Build Environ.

[CR88] Staiger H, Laschewski G, Grätz A (2012). The perceived temperature -- a versatile index for the assessment of the human thermal environment. Part A: scientific basics. Int J Biometeorol.

[CR89] Thorsson S, Tsuyoshi H, Lindberg F, Eliasson I, Lim E-M (2007) Thermal comfort and outdoor activity in Japanese urban public places. Environ Behav 39

[CR90] Tsitoura M, Tsoutsos T, Daras T (2014). Evaluation of comfort conditions in urban open spaces. Application in the island of Crete. Energy Convers Manag.

[CR91] VDI (2008a) Methods and presentation of investigations relevant for planning urban climate. VDI 3785

[CR92] VDI (2008b) Methods for the human biometeorological evaluation of climate and air quality for urban and regional planning at regional level, part I: Climate. VDI 3787

[CR93] VDI (2017). Environmental meteorology - prognostic microscale wind field models - evaluation for flow around buildings and obstacles.

[CR94] von Szombathely M (2017). A conceptual modeling approach to health-related urban well-being. Urban Sci.

[CR95] Walgama C, Fackrell S, Karimi M, Fartaj A, Rankin GW (2006). Passenger thermal comfort in vehicles - a review. Proceedings of the Institution of Mechanical Engineers, Part D: Journal of Automobile Engineering.

[CR96] Watanabe S, Nagano K, Ishii J, Horikoshi T (2014). Evaluation of outdoor thermal comfort in sunlight, building shade, and pergola shade during summer in a humid subtropical region. Build Environ.

