# Supplementary material for: Evaluation of thermal indices for their applicability in obstacle-resolving meteorology models
Source: Int J Biometeorol. 2018 Aug 14;62(10):1887–900. doi: 10.1007/s00484-018-1591-6 (PMC6153498; doi:10.1007/s00484-018-1591-6)
Supplement: Supplementary file 1 — (PDF 839 kb) [file 484_2018_1591_MOESM1_ESM.pdf]

Appendix A Indices excluded by Criteria C1-C7

Table 1 shows the indices excluded by C1 to C7, their abbreviation, references and the reason for their exclusion.

Table 1: Indices excluded from further analysis with the first criterion (C) they do not meet. Indices are given with their abbreviations (Abbr.) and reference in alphabetical order per failed criterion. Reasons for exclusion and comments include equations for the calculation of the indices if they are short enough. Indices for which differences are found in our literature review and the one by de Freitas and Grigorieva (2016) are marked with a star (*). Details on the differences are given in the Appendix. The air temperature design range of indices ($\boldsymbol{\Delta T}$) are taken from de Freitas and Grigorieva (2016). The following abbreviations of human body related parameter are used: $\boldsymbol{clo}$ is clothing, $\boldsymbol{E_{sk}}$ is evaporative heat loss from skin surface, $\boldsymbol{HR}$ is heart rate, $\boldsymbol{HB}$ is heart beats, $\boldsymbol{M}$ is metabolic heat, $\boldsymbol{PEx}$ is physical exertion, $\boldsymbol{R}$ is thermal resistance of clothing, $\boldsymbol{SR}$ is sweat rate, $\boldsymbol{T_b}$ is body temperature, $\boldsymbol{T_{cr}}$ is core temperature, $\boldsymbol{T_{rect}}$ is rectal temperature, $\boldsymbol{T_{sk}}$ is skin temperature, $\boldsymbol{T_{sk,init}}$ is initial skin temperature, $\boldsymbol{TS}$ is thermal sensation, $\boldsymbol{WL}$ is water loss. Additional parameters: $\boldsymbol{a}$ is a general function $\boldsymbol{e}$ is water vapour pressure, $\boldsymbol{e_s}$ is saturation water vapor pressure, $\boldsymbol{f}$ is vapor tension of air, $\boldsymbol{F}$ is vapor tension at 36.5 °C [mmHg] , $\boldsymbol{h}$ is hour of the day, $\boldsymbol{h_c}$ is convective heat transfer coefficient, $\boldsymbol{L}$ is longwave radiation, $\boldsymbol{n}$ is elevation, $\boldsymbol{N}$ is cloudiness, $\boldsymbol{p}$ is pressure, $\boldsymbol{P}$ is precipitation, $\boldsymbol{p_d}$ is diurnal pressure range, $\boldsymbol{q}$ is absolute humidity, $\boldsymbol{S}$ is solar radiation, $\boldsymbol{T}$ is air temperature, $\boldsymbol{T_d}$ is diurnal temperature range, $\boldsymbol{T_{dp}}$ is dew-point temperature, $\boldsymbol{T_g}$ is globe temperature,$\boldsymbol{T_{gr}}$ is ground temperature, $\boldsymbol{T_w}$ is mean temperature of surroundings, $\boldsymbol{T_{wall}}$ is wall temperature, $\boldsymbol{T_{wb}}$ is wet-bulb temperature, $\boldsymbol{Tu}$ is turbulence intensity, $\boldsymbol{v}$ is wind speed.

C	Index	Abbr.	Reference	Reason / Comments
1	Air Cooling Power	ACP	McPherson (1992)	Requires T_{sk}
1	Cold strain Index	CSI	Moran et al. (1999)	Requires T_{cr}, T_{sk}
1	Cumulative Heat Strain index	CHSI	Frank et al. (1996)	Requires HB, HR, T_{rect}
1	Grade of Heat strain	GHSI	Hubac et al. (1989)	Requires HR
1	Heat tolerance index	HTI	Hori (1978)	Requires T_{rect}, salt loss, WL
1	Increment Temperature Equivalent to Radiation Load	ITER	Lee and Vaughan (1964)	Requires SR
1	Index of Physiological Effect	E_P	Robinson et al. (1944)	Requires HR, T_{sk}, T_{rect}, SR
1	Maximum Exposure Time	MET_B	Brauner and Shacham (1995)	Requires $T_{sk,init}$
1	Perceptual Hyperthermia Index*	PHI	Gallagher et al. (2012)	Requires TS, PEx or T_c
1	Perceptual strain index*	PeSI	Tikuisis et al. (2002)	Requires TS, PEx
1	Physiological index of Strain	Is	Hall and Polte (1960)	Requires HR, T_{rect}, SR
1	Physiological Strain Index	PSI	Moran et al. (1998)	Requires HR, T_{rect}
1	Q_S-index (correct name: ΔQ_d-index, see Table 6)*		Rublack et al. (1981)	Requires T_{sk}
1	Quotient of heat stress	$Q_{dif,H}$	Hubac et al. (1989)	Requires HR
1	Skin Temperature	SKT	Mehnert et al. (2000)	Requires T_{rect}
1	Skin wettedness	SkW	Gonzalez et al. (1978)	Requires E_{sk}/ in original publication measurements were used. However, E_{sk} could be estimated from thermophysiological models (e.g. Gagge et al. (1986)) including all six variables. Nonetheless the index characterizes stress only for warm conditions and is thus rejected due to C7
1	Required Clothing Insulation	I_{req}	Holmer (1988)	Requires T_{sk} and SR / Except for minimum I_{req} ($I_{req,min}$), which is calculated for $T_{sk} = 30$ °C and

C	Index	Abbr.	Reference	Reason / Comments
				$SR = 0.06$. However, design range ($-35 \leq \Delta T \leq 10$) is smaller than required (rejected due to C7)
2	Climate Index	CI	Becker (2000)	Requires monthly averages of hot and cold days estimated from Predicted Mean Vote values
2	Heat Stress Index	HSI_{WK}	Watts and Kalkstein (2004)	Requires, among others, daily maximum and minimum Apparent Temperature values and numbers of consecutive days of heat stress
2	Mahoney scale	MS	Koenigsberger et al. (1971)	Requires monthly mean air temperature and humidity to estimate daytime and nighttime thermal stress
2	Spatial Synoptic Classification	SSC	Kalkstein and Nichols (1996); Sheridan (2002)	Requires long-term input (about 30-year) to determine seed days for weather classification
2	Summer Severity Index	SSI / I_o	McLaughlin and Shulman (1977)	Requires, among others, air temperature deviations from a 30-year average period
2	Weather Stress Index	WSI	Kalkstein and Valimont (1986)	Requires deviations from 40-year average of Apparent Temperature
3	Black sphere actinograph		Poschmann cited by Brüner (1959)	No fitted equation
3	Classification of Weather in Moments	CWM/ KPM	Golovina and Rusanov (1993)	No fitted equation / Table to read weather classification from T, RH, N, v
3	Comfort Index	CI	Terjung (1966); Terjung (1968)	No fitted equation / Only available as nomogram
3	Corrected Effective Temperature	CET	Bedford (1964)	No fitted equation / Only available as nomogram
3	Cylinder		Brown and Gillespie (1986)	No fitted equation
3	Daily Weather Types	DWT	Lecha Estela (1998)	No fitted equation / Table to read weather classification from T, e, N, P
3	Ellipsoid Index		Blazejczyk et al. (1998)	No fitted equation
3	Eupathescope		Brüner (1959); Dufton (1929)	No fitted equation
3	Evans Scale	ES	Evans (1980)	No fitted equation / Table to read comfort conditions from T, RH; comfort ranges derived from v, M, clo
3	Frigorimeter		Thilenius and Dorno (1925)	No fitted equation
3	Metal man (Thermal manikin)		Pedersen (1948) cited by Brüner (1959)	No fitted equation
3	Modified Effective Temperature	MET_S	Smith (1952)	No fitted equation / Only available as nomogram
3	Resultant thermometer		Missenard (1935) cited by Brüner (1959)	No fitted equation
3	Thermal Resistance of Clothing	TRC / $R_{t,wa}$	Jokl (1982)	If $T \neq T_{mrt}$, h_c must be read from a diagram. Otherwise TRC is only a function of v and the number of clothing layer (rejected due to C5)
3	Thermo-integrator		Winslow et al. (1935)	No fitted equation

C	Index	Abbr.	Reference	Reason / Comments
3	Effective Temperature	ET	Houghten and Yagloglou (1923) cited by Givoni (1976)	No fitted equation / Only available as nomogram
3	Heat Tolerance Limits	HTL	Vogt et al. (1982)	No fitted equation / Only available as nomogram
3	Mean Equivalence Lines	MEL	Wenzel (1978)	No fitted equation / Only available as nomogram
3	Predicted four hour sweat rate	P4SR	McArdle et al. (1947)	No fitted equation / Basic four hour sweat rate (input of P4SR) only available as nomogram
3	Still Shade Temperature	SST	Burton and Edholm (1955); Parsons (2014)	No fitted equation / The insulation decrement is only available in a table
3	Wind Effect Index	WEI	Terjung (1966)	No fitted equation / Only available as nomogram
4	Acclimatization Thermal Strain Index	ATSI	de Freitas and Grigorieva (2009)	Thermal stress due to abrupt change of climates / $ATSI = 100(Q_{rh} - Q'_r)/Q_{rh}$ Q_{rh} is respiratory heat loss at home and Q'_r at destination
4	Adaptation Strain index	ASI	Blazejczyk and Vinogradowa (2014)	Thermal stress due to abrupt change of climates
4	Bioclimatic Contrast Index	BCI	Blazejczyk (2011)	Thermal stress due to abrupt change of climates / $BCI = (\Delta UTCI + \Delta PST + \Delta WL + \Delta I_{clp})/4$ for parameter names see this table
4	Bioclimatic Distance Index	BDI	Mateeva and Filipov (2003) cited by Blazejczyk (2011)	Thermal stress due to abrupt change of climates / $BDI = (ECI_h - ECI)/13 \cdot 100$ ECI is effective clothing insulation, h indicates home location
4	Integral Load Index	ILI	Matyukhin and Kushnirenko (1986)	Thermal stress due to abrupt change of climates / methodology can be used for different meteorological parameters
4	Weather-Climate-Contrasts	WCC	Rusanov (1987)	Thermal stress due to abrupt change of climates / difference in clo-units between two climates in relation to maximum difference
5	Air Enthalpy	AirE i	Gregorczuk (1968)	Does not consider all 6 variables / $i = 0.24\left(T_{wb} + \frac{1.555}{p}\, e\right)$
5	Air temperature		MacPherson (1962)	Does not consider all 6 variables / Considers T
5	Apparent Temperature Atrocity of weather	AT	Arnoldy (1962)	Does not consider all 6 variables / Considers T, v
5	Apparent Temperature* or Heat Index	AT/ HI	Steadman (1979); Steadman (1984)	Does not consider all 6 variables / Considers T, e, v, S, M, Clo
5	Belgian Effective Temperature	BET TEL	Bidlot and Ledent (1947) cited by Brüner (1959); Eissing (1995)	Does not consider all 6 variables / $TEL = 0.9\, T_{wb}[°C] + 0.1\, T[°C]$
5	Bioclimatic Index of the Severity of Climatic Regime	BISCR	Belkin (1992)	Does not consider all 6 variables / Considers T, p, v, RH, n
5	Biometeorological	BCI	Rodriguez et al. (1985)	Does not consider all 6 variables /

C	Index	Abbr.	Reference	Reason / Comments
	Comfort Index			$BCI = \frac{t_a + T_{wb}}{2}$ $t_a = t_a(T_b, T, v)$
5	Bodman's Weather Severity Index	BWSI/ S	Bodman (1908)	Does not consider all 6 variables / $S = \frac{k(T,v)}{k(T_o,v_o)} = \frac{506(1-0.04T)(1+0.272v)}{506}$ Heat loss for specific situation $k(T,v)$ compared to reference situation $k(T_o,v_o)$; usually $T_o = 0$ °C, $v_0 = 0$ m/s
5	Body-atmosphere Energy Exchange Index	BIODEX	de Freitas and Ryken (1989)	Does not consider all 6 variables / Considers T, e, v, S, M, Clo
5	Clothing Insulation	I_c	Mount and Brown (1985)	Does not consider all 6 variables / Considers T,v, S, N, P
5	Clothing Thickness	Clo	Steadman (1971)	Does not consider all 6 variables / Considers T, v, S
5	Comfort Chart	CmCh	Mochida (1979)	Does not consider all 6 variables / Considers T, e, v, L, Clo, M Calculates T_{mrt} from surrounding walls
5	Comfort Vote	CmV S	Bedford (1936); Bedford (1961)	Does not consider all 6 variables / $S = 11.16 - 0.0556\, T[°\mathrm{F}] - 0.538\, T_g[°\mathrm{F}] - 0.0372\, e[\mathrm{mmHg}] + 0.00144\, v^{0.5}\left[\frac{\mathrm{ft}}{\mathrm{min}}\right](100 - T[°\mathrm{F}])$ From questionnaires in winter season in Great Britain for sedentary activity, only indoors
5	Cumulative Discomfort Index	CumDI	Tennenbaum et al. (1961)	Does not consider all 6 variables / $\sum_{h=1}^{h_{end}} \frac{T(h) - T_{wb}(h)}{2} - 24$ Hourly summation over period
5	Dew point temperature		Bruce (1916) cited by Brüner (1959); Eissing (1995)	Does not consider all 6 variables / Considers T_{dp}
5	Discomfort Index	DI_K	Kawamura (1965) cited by Ono and Kawamura (1991)	Does not consider all 6 variables / $DI_K = 0.99\, T[°\mathrm{C}] + 0.36 T_{dp}[°\mathrm{C}] + 41.5$ Based on DI_T
5	Discomfort Index or Temperature Humidity Index	DI_T / THI	Thom (1957) and Thom (1958) cited by Landsberg (1972); Tromp (1966)	Does not consider all 6 variables / $THI = T[°\mathrm{F}] - (0.55 - 0.55RH)(T[°\mathrm{F}] - 58)$ $DI_T = 0.4(T[°\mathrm{F}] + T_{wb}[°\mathrm{F}]) + 15$ $DI_T = 0.4(T[°\mathrm{C}] + T_{wb}[°\mathrm{C}]) + 4.8$
5	Draught Risk Index* / Percent dissatisfied	PD	Fanger et al. (1988)	Does not consider all 6 variables / $PD = 3.143(34 - T) \cdot (v - 0.05)^{0.6223} + 0.3696v \cdot Tu(34 - T)(v - 0.05)^{0.6223}$
5	Effective Temperature	ET_M	Missenard (1933) cited by Gregorczuk and Cena (1967)	Does not consider all 6 variables /

C	Index	Abbr.	Reference	Reason / Comments
				$ET = T[°C] - 0.4(T[°C] - 10)\left(1 - \frac{RH}{100}\right)$
5	Environmental Stress Index	ESI	Moran et al. (2001)	Does not consider all 6 variables / $ESI = 0.63T - 0.03RH + 0.002S + 0.0054(T \cdot RH) - 0.073(0.1 + S)^{-1}$
5	Equatorial Comfort Index or Singapore Index	ECI	Webb (1959)	Does not consider all 6 variables / $ECI = 0.574\,T + 0.488e - 0.231v^{0.5} + 21.23$ Sensations for Singapore climates indoors
5	Equivalent Effective Temperature	EET	Aizenshtat and Aizenshtat (1974)	Does not consider all 6 variables / $EET = T[1 - 0.003(100 - RH)] - 0.385v^{0.59}\,[(36.6 - T) + 0.662(v - 1)] + (0.0015v + 0.0008)\,(36.6 - T) - 0.0167]\,(100 - RH)$
5	Equivalent Rectal Temperature	ERT / T_{rec}	Givoni and Goldman (1972)	Does not consider all 6 variables / Considers T, e, v, M, Clo
5	Equivalent Temperature*	EqT	Bedford (1936); Bedford (1951)	Does not consider all 6 variables / $EqT = 0.522\,T\,[°F] + 0.478\,T_{mrt}[°F] - 0.01474\,\sqrt{v}\left[\frac{\text{ft}}{\text{min}}\right]\,(100 - T[°F])$ T_{mrt} from T_g or Eupatheoscope
5	Equivalent Warmth*	EqW	Bedford (1936)	Does not consider all 6 variables / $EqT = 0.522\,T\,[°F] + 0.478\,T_{mrt}[°F] - 0.01474\,\sqrt{v}\left[\frac{\text{ft}}{\text{min}}\right]\,(100 - T[°F])$ T_{mrt} from T_g
5	Exposed skin Temperature*	EST	Brauner and Shacham (1995)	Does not consider all 6 variables / Considers T, v, S
5	Globe Thermometer Temperature	T_g	Dimiceli et al. (2011); Vernon and Warner (1932)	Does not consider all 6 variables / Considers T_g, or in approximation equation T,v,e, S
5	Heart Rate Index	HRI_G	Givoni and Goldman (1973)	Does not consider all 6 variables / Considers T, e, v, M, Clo
5	Heat Stress Index*	HSI_{BH}	Belding and Hatch (1955)	Does not consider all 6 variables / Does not explicitly account for solar radiation in the equation for radiative balance.
5	Heat Stress Prediction Model / Heat Strain Model	HSPM/ ARIEM	Cadarette et al. (1999); Pandolf et al. (1985)	Does not consider all 6 variables / Considers T, e, v, S, M, Clo Different versions for laptop, pocket calculator and desktop exist. Based on HRI_G and T_{rec}
5	Humidex	HD	Masterson and Richardson (1979)	Does not consider all 6 variables / $HD = T\,[°C] + \frac{5}{9}(e[\text{mbar}] - 10)$
5	Humisery		Weiss (1982)	Does not consider all 6 variables / Humisery= $T + a(T_{dp}, v, n)$
5	Humiture		Pepi (1999); Weiss (1982)	Does not consider all 6 variables / Humiture= $T + T_{dp} - 18[°C]$

C	Index	Abbr.	Reference	Reason / Comments
				Humiture= $\frac{T+T_{dp}}{2}$<br>Humiture $= T[°\text{F}] + e[\text{mbar}] - 10[°\text{F}]$<br>Different versions exist
5	Index of Clothing required for Comfort*	CLODEX	de Freitas (1986); de Freitas (1987)	Does not consider all 6 variables / $CLODEX = \frac{T_s - T}{H} - \frac{I_a(H+S)}{H}$<br>with $T_s = 33$ °C, $H = 0.75\ M$ and $1/I_a = [0.61 + 0.19(v \cdot 100)^{0.5}]H$
5	Index of Pathogenicity of Meteorological Environment	IPME	Latyshev and Boksha (1965) cited by Kobyscheva et al. (2008)	Does not consider all 6 variables / Considers T, T_d, e, v, n, S, p_d
5	Index of Sultriness Intensity	ISI	Aikimovich and Balalla (1971)	Does not consider all 6 variables / Classes of e only
5	Index of thermal sensation	ITSN	Rohles and Nevis (1971)	Does not consider all 6 variables / Considers T, RH<br>Further developments link sensations also to new ET* and v Rohles et al. (1975); Rohles et al. (1974)
5	Index of thermal stress*	ITS_{GIV}	Givoni (1976)	Does not consider all 6 variables / L is not considered
5	Index of thermal stress	ITS_{KN}	Kondratyev (1957) cited by Rusanov (1981)	Does not consider all 6 variables / $N = \frac{0.16\ (T_{sk} - T)}{\frac{R}{0.175} + \frac{5.7}{a(v)}}$<br>$N = 0.78 \frac{M}{100}$
5	Insulation Predicted index*	I_{clp}	Blazejczyk (2011)	Does not consider all 6 variables / $I_{clp} = 0.082 \cdot \frac{[91.4 - (1.8 \cdot T + 32)]}{2.3274} - [1/0.61 + 1.9 \cdot v^{0.5}]$
5	Integral Index of Cooling Conditions	IICC	Afanasieva et al. (2009)	Does not consider all 6 variables / $IICC = 73.882 - 0.60361T + 1.3096v - 9.1985I_c - 0.15527M$
5	Kata thermometer		Hill and Hargood-Ash (1919); Maloney and Forbes (2011)	Does not consider all 6 variables / Approximation equations considers T, v, RH, S
5	Maximum Recommended Duration of Exercise*	MRDE	Young (1979)	Does not consider all 6 variables / Considers T, RH, S and Clo, M
5	Meteorological Health Index	MHI	Bogatkin and Tarakanov (2006)	Does not consider all 6 variables / Considers $T, RH, v, N, P, p, T_d, p_d$
5	Modified Discomfort Index	MDI	Moran et al. (2001)	Does not consider all 6 variables / $MDI = 0.75T_{wb} + 0.3T$
5	Modified (Reduced) Temperature / Equivalent facial skin temperatures*	MTTR / $T_{пр}$	Adamenko and Khairullin (1972)	Does not consider all 6 variables / Considers T, v
5	Natural Wet Bulb Temperature	NWBT T_n	Maloney and Forbes (2011)	Does not consider all 6 variables / $T_n = 0.85T + 0.17RH - 0.61v^{0.5}0.0016S - 11.62$
5	New Wind Chill	NWCI /	Office of the Federal	Does not consider all 6 variables /

C	Index	Abbr.	Reference	Reason / Comments
	Temperature Index	WCET / WCI	Coordinator for Meteorological services and supporting research (2003); Osczevski and Bluestein (2005)	$WCT[°C] = 13.12 + 0.6215T[°C] - 11.37v^{0.16}[km/h] + 0.3965v^{0.16}[km/h]$
5	Oxford Index / Wet-Dry Index*	OxI WD	Lind et al. (1956) cited by Bedford (1957); Lind and Hellon (1957)	Does not consider all 6 variables / $WD = 0.15\,T + 0.85T_{wb}$
5	Operative Temperature	OpT T_o	Winslow and Herrington (1949); Winslow et al. (1937)	Does not consider all 6 variables / Summarizes effect of dry heat exchange; Considers T, v, T_{mrt} in original form T_{wall}
5	Outdoor Apparent Temperature	OAT	Steadman (1984); Steadman (1994)	Does not consider all 6 variables / Considers T, e, v, S, M, Clo; regression version is more frequently used than complete model version
5	Physiological Heat Exposure Limit Chart	PHEL	Dasler (1977)	Does not consider all 6 variables / Considers time-weighted-mean of WBGT and M
5	Radiation Equivalent Effective Temperature	REET	Sheleihovskyi (1948) cited by Rusanov (1981)	Does not consider all 6 variables / Considers T, e, v, S
5	Relative Heat Strain*	RHS	Lee and Henschel (1966)	Does not consider all 6 variables / Considers T, e, v, L and Clo, M
5	Relative Humidity Dry Temperature	RHDT	Wallace et al. (2005)	Does not consider all 6 variables / $RHDT = 0.9T + 0.1RH$
5	Respiratory Heat Loss	RHL/ Q_R	Rusanov (1989) cited by de Freitas and Grigorieva (2016)	Does not consider all 6 variables / C1 to C4 not checked since required literature could not be obtained. Considers T, e, p, el, M
5	Resultant Temperature or Net Effective Temperature	RT/ NET	Missenard cited by Landsberg (1972)	Does not consider all 6 variables / $NET = 37 - (37 - T) \cdot \left(0.68 - 0.0014RH + \frac{1}{1.76+1.4v^{0.75}}\right)^{-1} - 0.29T\left(1 - \frac{RH}{100}\right)$
5	Saturation deficit		Flügge (1912) cited by Brüner (1959)	Does not consider all 6 variables / Considers q
5	Severity Rating	S	Osokin (1968) cited by Rusanov (1981)	Does not consider all 6 variables / $S = (1 - 0.06T)(1 + 0.20v)(1 + 0.0006n)K_b(RH)A_c(T_d)$
5	Standard Operative Temperature	To'/ T_{SO}	Gagge et al. (1973)	Does not consider all 6 variables / Considers T, v, T_{mrt}; T_{sk} can be calculated from provided model
5	Subjective Temperature	T_{sub}	McIntyre (1973)	Does not consider all 6 variables / $T_{sub} = 0.44\,T_r + \frac{0.56\left(5 - \sqrt{10v}(5 - T)\right)}{0.44 + 0.56\sqrt{10v}}$
5	Summer Simmer Index	SSI	Pepi (1987); Pepi (1999); Tzenkova et al. (2007)	Does not consider all 6 variables / $SSI = T[°F] - (0.55 - 0.0055 \cdot RH[\%]) \cdot (T\,[°F] - 58)) - 56.83$ Different versions exist (further developments)
5	Sultriness value		Scharlau (1943)	Does not consider all 6 variables /

C	Index	Abbr.	Reference	Reason / Comments
				Considers e
5	Survival Time Outdoors in Extreme Cold*	STOEC	de Freitas and Symon (1987)	Does not consider all 6 variables / Considers T, e, v, S, Clo, M
5	Temperature Humidity Index	THI_S	Schoen (2005)	Does not consider all 6 variables / $THI = T - 1.0799e^{0.03755T}(1 - e^{0.0801(T_{dp}-14)})$
5	Temperature-Wind Speed-Humidity Index	TWH	Zaninovic (1992)	Does not consider all 6 variables / Considers T, v, e_s
5	Thermal Acceptance Ratio	TAR	Ionides, Plummer and Siple (1945) cited by Auliciems and Szokolay (2007)	Does not consider all 6 variables / Considers T, e, L, M
5	Thermal Balance	$ThBal_r$ / Q_S	Rusanov (1981)	Does not consider all 6 variables / 2 versions exist: full heat balance version that includes all terms ($ThBal_b$, Table 2) and a regression version based on EET, which does not consider longwave radiation and is applicable only for nude persons ($ThBal_r$) but has an assessment scale
5	Thermal Insulation Characteristics of Clothing	TICC / R	Kondratyev (1957) cited by Rusanov (1981)	Does not consider all 6 variables / $R = 3.36\frac{T_{sk} - T}{M} - \frac{0.99}{a(v)}$ T_{sk} set to 33 °C
5	Thermal Insulation of Clothing	TIC_B	Budyko and Cicenko (1960); Liopo and Cicenko (1971)	Does not consider all 6 variables / Regression equation considering T, v, S and M fitted by Liopo and Cicenko (1971) to full heat balance equation by Budyko and Cicenko (1960) and related derived nomograms
5	Thermal Insulation of Clothing	TIC_R	Rusanov (1981)	Does not consider all 6 variables / Is based on $ThBal_r$ and therefore does not consider longwave radiation
5	Thermal Insulation of Protective Clothing	TIPC	Afanasieva (1977)	Does not consider all 6 variables / Considers T, v, M. Designed especially for winter conditions (S-input is assumed very small)
5	Thermal Sensation Index*	TSNI	de Paula Xavier and Lamberts (2000)	Does not consider all 6 variables / Regression equation developed for indoors; coefficient of T_o is probably different if solar radiation is included. $S = 0.219\,T_o + 0.012RH - 0.547v - 5.83$
5	Thermal Strain Index	TSI / G	Lee (1958)	Does not consider all 6 variables / $G = a\left[\frac{(M-W) - \frac{5.55(34-T)}{I_a(v)+I_c} - 0.00033(46-e)}{\frac{c-e}{r_a(v)+r_c}}\right]^d$
5	Total Thermal Stress*	TTS	Auliciems and Kalma (1981)	Does not consider all 6 variables / Does not consider L
5	Tropical summer index	Tsi	Bureau of Indian	Does not consider all 6 variables /

C	Index	Abbr.	Reference	Reason / Comments
			Standards (1987) cited by Auliciems and Kalma (1981)	$Tsi = 0.308T_{wb} + 0.745\,T_g - 2.06\sqrt{v + 0.841}$
5	Wet Bulb Dry Temperature	WBDT	Wallace et al. (2005)	Does not consider all 6 variables / $WBGT = 0.4T_{wb} + 0.6T$
5	Wet Bulb Globe Temperature	WBGT	Auliciems and Kalma (1981); Yaglou and Minard (1957)	Does not consider all 6 variables / $WBGT = 0.7T_{wb} + 0.2T_g + 0.1T$
5	Wet Bulb Temperature	T_{wb}	Brüner (1959); Eissing (1995); Stull (2011)	Does not consider all 6 variables / Approximation equation considers T, RH
5	Wet Kata Cooling Power by Hill	WKCP H_w	Hill and Hargood-Ash (1919)	Does not consider all 6 variables / $H = (0.27 + 0.49\sqrt{v})(36.5 - T) + (0.85 + 0.102v^{0.3})(F - f)^{4/3}$
5	Wind Chill Equivalent Temperature	WCT_{wc} Twc	Falconer (1968)	Does not consider all 6 variables / $Twc\ [°F] \approx -(\sqrt{v \cdot 100} + 10.45 - v)\,(91.4 - T[F]) \cdot (\sqrt{1.34 \cdot 100} + 10.45 - 1.34) + 91.4)^{-1}$ Under sunshine cooling is reduced
5	Wind Chill Equivalent Temperature	WCET	Steadman (1971)	Does not consider all 6 variables / Considers T, v, L, M, I_{cl}; L, M and I_{cl} are assumed fixed
5	Wind Chill Index	WCI	Siple and Passel (1945)	Does not consider all 6 variables / Considers T,v
6	Thermal Sensation	TS_{GIV}	Givoni et al. (2003)	Does not consider longwave radiation from all directions / $TS_{GIV} = 1.7 + 0.1118T + 0.0019S - 0.322v - 0.0073RH + 0.0054T_{gr}$ For fixed clothing + activity; considers only longwave radiation from ground
7	Body Temperature Index	BTI	Dayal (1974)	Air temperature range smaller than -5 °C to 35 °C / Designed for $30 \leq \Delta T \leq 42$; Equation for T_{mrt} from T_g-measurements might be needed to be adapted to consider solar influence
7	Effective Heat Strain Index	EHSI	Kamon and Ryan (1981)	Air temperature range smaller than -5 °C to 35 °C / Designed for $27 \leq \Delta T \leq 36$; Equation for T_{mrt} from T_g-measurements might be needed to be adapted to consider solar influence
7	Heart Rate Index	HRI_D	Dayal (1974)	Air temperature range smaller than -5 °C to 35 °C / Designed for $30 \leq \Delta T \leq 42$; Equation for T_{mrt} from T_g-measurements might be needed to be adapted to consider solar

C	Index	Abbr.	Reference	Reason / Comments
				influence
7	Heat Strain Decision Aid Model	HSDA	Cadarette et al. (1999); Santee and Wallace (2003)	Air temperature range smaller than -5 °C to 35 °C / Designed for $18 \leq \Delta T \leq 43$
7	Humid Operative Temperature	HToh / T_{oh}	Gagge et al. (1973); Gagge et al. (1971)	Air temperature range smaller than -5 °C to 35 °C / Designed for $10 \leq \Delta T \leq 40$
7	New Effective Temperature	ET*	Gagge et al. (1973); Gagge et al. (1971)	Air temperature range smaller than -5 °C to 35 °C / Designed for $10 \leq \Delta T \leq 40$
7	Predicted Mean Vote – indoors	PMV	Fanger (1970)	Temperature range smaller than -5 °C to 35 °C / Designed for $15 \leq \Delta T \leq 45$ [indoors]
7	Predicted Mean Vote – outdoors*	PMV*	Gagge et al. (1986)	Air temperature range smaller than -5 °C to 35 °C / Designed for $0 \leq \Delta T \leq 50$
7	Predicted Mean Vote – Fuzzy	PMV_F	Hamdi et al. (1999)	Air temperature range smaller than -5 °C to 35 °C / Designed for $-10 \leq \Delta T \leq 32$; Fuzzy logical estimation of PMV. Designed for indoors; Rules for T_{mrt} may require adjustment if used outdoors
7	Predicted Percentage Dissatisfied	PPD	ASHRAE (2001); Fanger (1970)	Air temperature range smaller than -5 °C to 35 °C / Designed for $15 \leq \Delta T \leq 45$ [indoors]
7	Reference Index	RI	Pulket et al. (1980)	Air temperature range smaller than -5 °C to 35 °C / Designed for $30 \leq \Delta T \leq 40$; Originally included only L; but expected to work if S is included as based on heat balance principles
7	Required Sweat Rate	Req SR / S_r	Vogt et al. (1981)	Air temperature range smaller than -5 °C to 35 °C / Designed for $20 \leq \Delta T \leq 60$
7	Standard Effective Temperature	SET*	Gagge et al. (1973); Gonzalez et al. (1974)	Air temperature range smaller than -5 °C to 35 °C / Designed for $0 \leq \Delta T \leq 50$
7	Thermal Discomfort	DISC	Gagge et al. (1986)	Air temperature range smaller than -5 °C to 35 °C / Designed for $10 \leq \Delta T \leq 50$; calculated from 2-node model
7	Thermal Work Limit	TWL	Brake and Bates (2002)	Air temperature range smaller than -5 °C to 35 °C / Designed for $36 \leq \Delta T \leq 40$; developed for indoors but uses heat balance equations with T_{mrt} so S can be included

Appendix B Found differences in index inputs

To evaluate the criteria for the different indices in Sec.3, the original publication of the indices were reviewed. For some indices our analysis of the indices differed from the results by de Freitas and Grigorieva (2016). This might be in some cases due to the use of secondary literature by de Freitas and Grigorieva (2016). In other cases we interpret the same publication differently, indicating that indices are not always thoroughly documented. The found differences of index characteristics are documented in Table 2. As evidence for our interpretation citations or equations are given.

Table 2: Index characteristics found in our literature review of the thermal indices and used in the present study compared to the ones by de Freitas and Grigorieva (2016). Atmosphere-related variable inputs are denoted “A” and body-related variable inputs are denoted “B”. The following abbreviations are used: $\boldsymbol{clo}$ is clothing, $\boldsymbol{e}$ is vapor pressure, $\boldsymbol{e_s}$ is saturation vapor pressure, $\boldsymbol{e_{s,sk}}$ is saturated water vapor pressure at $\boldsymbol{T_{sk}}$, $\boldsymbol{E_{sk}}$ is evaporative heat loss from skin surface, $\boldsymbol{HR}$ is heart rate, $\boldsymbol{I_{cl}}$ is clothing insulation, $\boldsymbol{L}$ is longwave radiation, $\boldsymbol{M}$ is metabolic rate, $\boldsymbol{PE}$ is physical exertion, $\boldsymbol{RH}$ is relative humidity, $\boldsymbol{r_b}$ is body tissue thermal resistance, $\boldsymbol{S}$ is solar radiation, $\boldsymbol{T}$ is air temperature, $\boldsymbol{T_c}$ is core temperature, $\boldsymbol{T_g}$ is globe temperature, $\boldsymbol{T_{gr}}$ is ground temperature, $\boldsymbol{T_{mrt}}$ is mean radiant temperature, $\boldsymbol{T_{sk}}$ is skin temperature, $\boldsymbol{T_w}$ is mean temp of surroundings, $\boldsymbol{T_{wb}}$ is wet bulb temperature, $\boldsymbol{TS}$ is thermal sensation, $\boldsymbol{Tu}$ is turbulence intensity, $\boldsymbol{v}$ is wind speed.

**Index (Abbreviation)**	**Variable inputs considered according to de Freitas and Grigorieva (2016) (cited reference)**	**Variable inputs considered according to our review (reference)**	**Evidence, Comments**
Apparent Temperature (AT) or Heat Index (HI)	A: T, e, S<br>B: Clo, M<br>(Steadman 1979; Steadman 1984)	A: T, e, v, S<br>B: No<br>(Steadman 1979; Steadman 1984)	Using the nomenclature of this paper the publication by Steadman (1984) reads: “The apparent temperature of a set of meteorological conditions T, e, v, S may be defined as equal to dry-bulb temperature at $v = S = 0$, and at a base vapor pressure of moderate humidity, which would require the same thermal resistance, in a walking adult, as this set of conditions”. Clothing and activity are considered in AT but fixed and are therefore no variable inputs. From the full model regression equations were developed, which are used far more frequently. In the final development stage (Steadman 1979) the scope of the index “has been enlarged to cover the range of dry-bulb temperatures from -40 to +50 °C”. This range is larger than +20 to +60 °C mentioned by de Freitas and Grigorieva (2016)
Draught Risk Index (PD; Percent dissatisfied)	A: T, v<br>B: No<br>(Fanger et al. 1988)	A: T, v, Tu<br>B: No<br>(Fanger et al. 1988)	The full equation reads:<br>$PD = 3.143(34 - T)(v - 0.05)^{0.6223} + 0.3696v \cdot Tu(34 - T)(v - 0.05)^{0.6223}$<br>Thus, turbulent intensity Tu is included as input.
Equivalent Temperature (EqT)	Not considered	A: T, v, T_w<br>B: No<br>(Bedford 1936; Bedford 1951)	EqT is mentioned by de Freitas and Grigorieva (2015) but not analyzed by de Freitas and Grigorieva (2016). The definition reads:<br>$EqT = 0.522\, T[°F] + 0.478\, T_w[°F] - 0.01474\, \sqrt{v}\, [ft/min](100 - T[°F])$

Index (Abbreviation)	Variable inputs considered according to de Freitas and Grigorieva (2016) (cited reference)	Variable inputs considered according to our review (reference)	Evidence, Comments
Equivalent Warmth (EqW)	A: T, T_{mrt}, e<br>B: T_{sk}<br>(Bedford (1936) cited by Auliciems and Szokolay (2007))	A: T, T_w, e_s, v<br>B: No<br>(Bedford 1936)	The definition is: $EqW = 9.979 - 0.1495\,x^2 - 2.89$<br>$x = 0.0556\,T + 0.0538\,T_w + 0.0372\,e_s - 0.00144\,\sqrt{v}(100 - T)$
Exposed skin Temperature (EST)	A: T, v, S<br>B: M<br>(Brauner and Shacham 1995)	A: T, v, S<br>B: No<br>(Brauner and Shacham 1995)	The equation reads:<br>$$\frac{T_c - T_s}{r_b} = \frac{T_c - T}{r_b + 1/h_c}$$<br>Fixed $M = 58$ Wm^{-2} (comfortable steady state condition) is used for calculating r_b: "The body tissue thermal resistance, r_b, can be estimated from Eq. 7 by introducing known values of thermal comfort in a normal temperature room […]. Under such conditions […], the metabolic heat production while sitting at rest is approximately equal to 50 kcal h^{-1}m^{-2} (58 Wm^{-2}), and […]. Thus, r_b is approximately 0.08 kca1^{-1} h °C m^2 […]." (Brauner and Shacham 1995)
Heat Stress Index (HSI_{BH})	A: T, T_g, e, v<br>B: Clo, M<br>(Belding and Hatch 1955)	A: T, T_g, e, v<br>B: M<br>(Belding and Hatch 1955)	"Clothing is the third variable fixed for the estimate, and it is unfortunate that limitations of available knowledge make it necessary to fix on a no-clothing basis." (Belding and Hatch 1955)
Index of Clothing Required for Comfort (CLODEX)	A: T, v, e, L, S<br>B: Clo, M<br>(de Freitas 1986; de Freitas 1987)	A: T, v, S<br>B: M<br>(de Freitas 1986; de Freitas 1987)	The definition is<br>$$CLODEX = \frac{T_s - T}{H} - \frac{I_a(H + S)}{H}$$<br>with $T_s = 33$ °C, $H = 0.75\,M$ and $1/I_a = [0.61 + 0.19(v \cdot 100)^{0.5}]H$. Thus, humidity and longwave radiation is not considered and clothing is not a variable input
Index of thermal Stress (ITS_{GIV} or I.T.S.)	A: T, e, v, S, L<br>B: Clo, M<br>(Givoni 1969)	A: T, e, v, S<br>B: Clo, M<br>(Givoni 1976)	"The I.T.S. does not as yet separately cover the factor of longwave radiation" (Givoni 1976)
Insulation Predicted index (I_{clp})	A: T, v<br>B: M<br>(Blazejczyk 2011)	A: T, v<br>B: No<br>(Blazejczyk 2011)	The definition is<br>$$I_{clp} = 0.082 \cdot [91.4 - (1.8 \cdot T + 32)]/2.3274 - [1/0.61 + 1.9 \cdot v^{0.5}]$$<br>Thus, no variable metabolic heat is considered
Maximum Recommended Duration of Exercise (MRDE)	A: T, e, S<br>B: M<br>(Young 1979)	A: T, RH, S<br>B: Clo, M<br>(Young 1979)	"The MRDE is determined by the level of exercise, the ambient temperature and humidity, the solar radiation and the clothing worn" (Young 1979)

Index (Abbreviation)	Variable inputs considered according to de Freitas and Grigorieva (2016) (cited reference)	Variable inputs considered according to our review (reference)	Evidence, Comments
Modified (Reduced) Temperature (MTTR, $T_{пр}$)	A: T, v, S<br>B: No<br>(Adamenko and Khairullin 1972)	Not found in cited reference, however for θ_{rf} cited in reference:<br>A: T, v<br>B: No<br>(Adamenko and Khairullin 1972)	In the publication cited by de Freitas and Grigorieva (2016) for the index MTTR no temperature termed Modified (Reduced) Temperature could be found. Instead an equivalent facial skin temperature (θ_{rf}) derived only from T and v is presented in the publication.
Oxford Index (OxI)/ Wet-Dry Index (WD)	A: T, T_{wb}<br>B: No<br>(Lind and Hellon 1957)	Not found in cited reference, however from secondary literature:<br>A: T, T_{wb}<br>B: No<br>(Lind et al. (1956) cited by Bedford (1957); Lind and Hellon (1957))	The cited publication is wrong: in the publication cited by de Freitas and Grigorieva (2016) for the Oxford Index no index termed Oxford Index or Wet-Dry Index could be found. However, from the book review by Bedford (1957) of "Lind A.R., Weiner J.S., Hellon R.F., Jones R.M., Fraser D.C. (1956) Reactions of Mines-Rescue Personal to Work in Hot Environments, Medical Research Memorandum No 1" the equation given in Table 1 could be retrieved and therefore the variable inputs could be confirmed.
Perceptual strain index (PeSI)	A: T, e<br>B: T_c, HR<br>(Tikuisis et al. 2002)	A: No<br>B: No<br>(Tikuisis et al. 2002)	The definition is<br>$$PeSI = 5 \cdot \frac{TS_t - 7}{6} + 5 \cdot \frac{PE_t}{10}$$<br>Thus, only thermal sensation and physical exertion are needed.
Perceptual Hyperthermia Index (PHI)	A: No<br>B: T_c, HR<br>(Gallagher et al. 2012)	A: No<br>B: T_c<br>(Gallagher et al. 2012)	"The development of the PHI consisted of calculating PeSI values for all RPE-RTS combinations. […] Next, the mean T_c coincident with each calculated PeSI value was determined. These T_c values subsequently replaced the PeSI values on the constructed figure therefore linking the perceptual variables of RPE and RTS with the physiological criterion of T_c." (Gallagher et al. 2012) Thus, PHI can be estimated either from TS and PE or from T_c. Heart rate was measured and found to be well correlated with TS and PE but is not further integrated into the calculation of PHI ranges.
Perceived Temperature (PT_L)	A: T, v, L<br>B: No<br>Linke (1926) cited by Eissing (1995)	Not found (Linke 1926)	In the publication cited by de Freitas and Grigorieva (2016) for PT_L no such index could be found. Instead an equation to calculate the heat input from radiation measured with a specific kind of a black globe thermometer is presented in the publication.

**Index (Abbreviation)**	**Variable inputs considered according to de Freitas and Grigorieva (2016) (cited reference)**	**Variable inputs considered according to our review (reference)**	**Evidence, Comments**
Physical saturation deficit	A: e<br>B: No<br>(Thilenius and Dorno (1925) cited by Eissing (1995))	Not found (Thilenius and Dorno 1925)	In the publication cited by de Freitas and Grigorieva (2016) for the index physical saturation deficit (Thilenius and Dorno (1925) cited by Eissing (1995)) the following definition is given "Difference between the vapour pressure of the ambient air and the vapour pressure of exhaled air". However in the original publication (Thilenius and Dorno 1925) no such index is described. Instead the Frigorimeter (Table 1) is described.
Relative Heat Strain (RHS)	A: T, T_{wb}, e, v<br>B: Clo, M<br>(Lee and Henschel 1966)	A: T, e, v, L<br>B: Clo, M<br>(Lee and Henschel 1966)	"The equation just cited includes terms for air temperature, humidity, air movement, radiant heat, metabolic rate and clothing"(Lee and Henschel 1966)
Skin wettedness (SkW, w)	A: T, T_w<br>B: No<br>(Gonzalez et al. 1978)	A: e<br>B: $E_{sk}, e_{s,sk}$<br>(Gonzalez et al. 1978)	"Skin wettedness (w), defined as the fraction of the subjects' body surface area covered by evaporative moisture, was determined as a ratio of the observed E_{sk} to maximum evaporation ($E_{\max}$) possible to the environment, assuming a subject's entire surface is completely wet."(Gonzalez et al. 1978)<br>$$w = \frac{E_{sk}}{E_{\max}} = \frac{E_{sk}}{h_e(e_{s,sk} - e)}$$<br>h_e is the evaporative heat transfer coefficient
Survival Time Outdoors in Extreme Cold (STOEC)	A: T, v, S<br>B: M<br>(de Freitas and Symon 1987)	A: T, e, v, S<br>B: M<br>(de Freitas and Symon 1987)	STOEC includes e to estimate respiratory heat loss (using the nomenclature of this paper):<br>$$E_{res} = 1.73 \cdot 10^{-3} M(44 - e)$$<br>Clothing is taken into account for convective heat exchange but fixed ($I_{cl} = 4$ clo).
Thermal Insulation of Clothing (TIC_A)	A: T, e, v, S, L<br>B: No<br>(Aizenshtat 1964)	Not found (Aizenshtat 1964)	In the publication cited by de Freitas and Grigorieva (2016) for the index TIC_A (Aizenshtat (1964)) no index TIC_A could be found. Instead this paper describes how a globe thermometer can be used to evaluate the thermal balance of a person.
Thermal Sensation Index (TSNI)	A: T, e, v, T_{mrt}<br>B: Clo, M<br>(de Paula Xavier and Lamberts 2000)	A: T, e, v, T_{mrt}<br>B: No<br>(de Paula Xavier and Lamberts 2000)	"The activity was constant (school activity) and not considered to be an independent variable influencing the sensation of thermal comfort. In our studies, we do not treat the thermal insulation of clothes as an independent variable but as dependent on the external temperature" (de Paula Xavier and Lamberts 2000):<br>$$S = 0.219\, T_o + 0.012RH - 0.547v - 5.83$$<br>Thus, clothing and metabolic heat are not variable inputs.
Total Thermal Stress (TTS)	A: T, e, v, S, L<br>B: No<br>(Auliciems and Kalma 1981)	A: T, e, v, S<br>B: No<br>(Auliciems and Kalma 1981)	"The net gain of shortwave solar radiation must be incorporated […]. $(Q+q)_m$ is the sum of net direct (Q) and diffuse (q) radiation falling upon man" (Auliciems and Kalma 1981). Includes only direct and diffuse radiation and no longwave radiation

**Index (Abbreviation)**	**Variable inputs considered according to de Freitas and Grigorieva (2016) (cited reference)**	**Variable inputs considered according to our review (reference)**	**Evidence, Comments**
Q_S-index<br>Correct name: ΔQ-index	A: T, e, v, L<br>B: Clo, M, T_{sk}<br>(Rublack et al. (1981) cited by Graveling et al. (1988))	A: T, e, v, L<br>B: Clo, M, T_{sk}<br>(Rublack et al. 1981)	The Q_S-index cited by Graveling et al. (1988) should be named Δq-index since Q_s according to the original publication (Rublack et al. 1981) describes only the longwave component in ΔQ:<br>$$\Delta Q = Q_M + Q_c + Q_s - Q_{v,\max}(e)$$

Appendix C Systematic literature review of thermal comfort studies with ORMs

A systematic literature review using the databases "Scopus" and "Web of Science" was conducted to identify which thermal indices have been used in the past with ORMs. Figure 1 shows the flow diagram corresponding to the method described in Sec. 2.4. Table 3 shows the 32 studies included in the analysis for F6 ordered by thermal index and climatic zone.

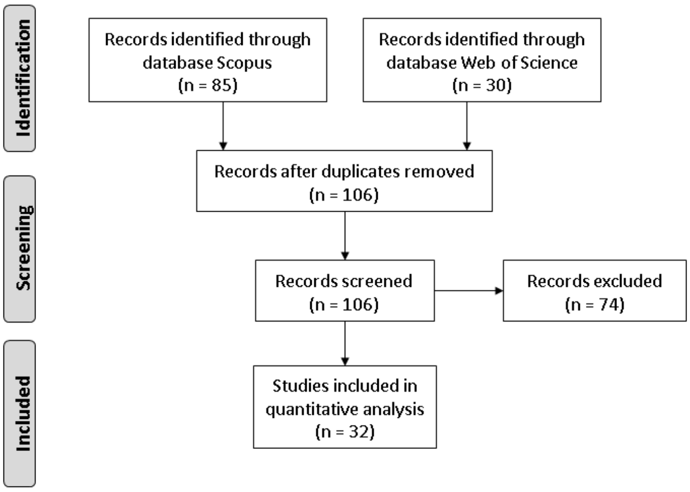

Figure 1: Flow Diagram for the systematic literature review adapted from the standardized Preferred Reporting Items for Systematic Reviews and Meta-Analysis (PRISMA) flow diagram (Moher et al. 2009) with changes.

Table 3: Cited studies to evaluate application frequency of indices. Studies have been selected according to the method in Sec. 2.4. For abbreviations of indices see Table 1 (Appendix A) and Table 1 (Sec. 3).

Index	Zone	References
PET	Tropics	Qaid et al. (2016); Morakinyo et al. (2016);
	Sub-tropics	Morakinyo and Lam (2016); Taleghani et al. (2016); Yang et al. (2015); Lopes et al. (2014); Yahia and Johansson (2014); Chen and Ng (2013); Peng and Jim (2013); Yang et al. (2011); Ali-Toudert and Mayer (2006)
	Mid-latitudes	Zölch et al. (2016); Lobaccaro and Acero (2015); Acero and Herranz-Pascual (2015); Taleghani et al. (2015); Ketterer and Matzarakis (2015); Ketterer and Matzarakis (2014); Müller et al. (2014); Ketterer et al. (2013); Minella et al. (2014)
PMV	Sub-tropics	Hedquist and Brazel (2014) (PMV); Stavrakakis et al. (2012) (PMV (extended version)); Zhang et al. (2012) (PMV (extended version))
	Mid-latitudes	Robitu et al. (2006) (PMV*)
SET*	Sub-tropics	He and Hoyano (2010) (OUT_SET*); He (2011) (OUT_SET*); Huang et al. (2005) (SET*)
THI	Tropics	Morakinyo et al. (2016); Kakon et al. (2009);
UTCI	Mid-latitudes	Goldberg et al. (2013); Schrijvers et al. (2016); Tumini et al. (2016); Park et al. (2014); Minella et al. (2014)
WBGT	Tropics	Morakinyo et al. (2016)

References

Acero JA, Herranz-Pascual K (2015) A comparison of thermal comfort conditions in four urban spaces by means of measurements and modelling techniques Building and Environment 93, Part 2:245-257 doi:http://dx.doi.org/10.1016/j.buildenv.2015.06.028

Adamenko VN, Khairullin KS (1972) Evaluation of conditions under which unprotected parts of the human body may freeze in urban air during winter Boundary-Layer Meteorology 2:510-518

Afanasieva R (1977) Hygienic theory of cold protection clothes projection. Legkaya Industriya. Moscow

Afanasieva R, Bobrov A, Sokolov S (2009) Cold Assessment Criteria and Prediction of Cooling Risk in Humans: The Russian Perspective Industrial Health 47:235-241

Aikimovich NN, Balalla OA (1971) Sultry weathers at the south of Primorye and their influence on human body Izvestia ASc USSR, Geography 4:94–100

Aizenshtat BA (1964) Methods for assessment of some bioclimate indices Meteorol Hydrol 12:9 - 16

Aizenshtat LB, Aizenshtat BA (1974) Equation for equivalent-effective temperature Questions of biometeorology, Hydrometeoizdat, Leningrad:81 - 83

Ali-Toudert F, Mayer H (2006) Numerical study on the effects of aspect ratio and orientation of an urban street canyon on outdoor thermal comfort in hot and dry climate Building and Environment 41:94 - 108 doi:https://doi.org/10.1016/j.buildenv.2005.01.013

Arnoldy IA (1962) Acclimatization of the man in north and south. Medgiz, Moscow

ASHRAE (2001) ASHRAE Handbook: Fundamentals. 8. American Society of Heating and Air-Conditioning Engineers,

Auliciems A, Kalma JD (1981) Human thermal climates of Australia Australian Geographical Studies 19:3 - 24

Auliciems A, Szokolay SV (2007) Thermal comfort. 2 edn. PLEA in association with Dept. of Architecture, University of Queensland Brisbane, Qld,

Becker S (2000) Bioclimatological Rating of Cities and Resorts In South Africa According to the Climate Index International Journal Of Climatology 20:1403 - 1414

Bedford T (1936) Warmth factor in comfort at work Med Res Council, Industrial Health Research Board, Report

Bedford T (1951) Equivalent Tempeature - What it is - how it's measured Heating, Piping & Air Conditioning 8:87 - 91

Bedford T (1957) Reactions of Mines-Rescue Personnel to Work in Hot Environments British Journal of Industrial Medicine 14:300

Bedford T (1961) Researches on thermal comfort, Given at Bristol, 17 April Ergonomics 4:289-310 doi:http://dx.doi.org/10.1080/00140136108930531

Bedford T (1964) Basic Principles of Ventilation and Heating. 2 edn. H.K. Lewis and Co, London, London

Belding HS, Hatch TF (1955) Index for Evaluating Heat Stress in Terms of Resulting Physiological Strains Heating, Piping & Air Conditioning 27:129 - 136

Belkin VS (1992) Biomedical Aspects of the Development of Mountain Regions: A Case-Study for the Gorno-Badakhshan Autonomous Region, Tajikistan Mountain Research and Development 12:63-70

Blazejczyk K (2011) Assessment Of Regional Bioclimatic Contrasts In Poland Miscellanea Geographica 15:79-91

Blazejczyk K, Homer I, Nilsson H (1998) Absorption of Solar Radiation by an Ellipsoid Sensor Simulated the Human Body Applied Human Science 17:267-273

Blazejczyk K, Vinogradowa V (2014) Adaptation Strain Index for tourists traveling from central and northern Europe to the Mediterranean Finisterra XLIX 98:139-152

Bodman G (1908) Das Klima als eine Funktion von Temperatur und Windgeschwindigkeit in ihrer Verbindung vol II. Lithographisches Institut des Generalstabs, Stockholm

Bogatkin OG, Tarakanov GG (2006) Basics of Meteorology (Основы метеорологии). St Petersburg

Brake DJ, Bates GP (2002) Limiting Metabolic Rate (Thermal Work Limit) as an Index of Thermal Stress Applied Occupational and Environmental Hygiene 17:176-186

Brauner N, Shacham M (1995) Meaningful wind chill indicators derived from heat transfer principles Int J Biometeorology 39:46-52

Brown RD, Gillespie TJ (1986) Estimating outdoor thermal comfort using a cylindrical radiation thermometer and an energy budget model International Journal of Biometeorology 30:43-52

Brüner H (1959) Arbeitsmöglichkeiten unter Tage bei erschwerten klimatischen Bedingungen Int Z angew Physiol einschl Arbeitsphysiol 18:31-61

Budyko M, Cicenko V (1960) Climatic factors of human thermal sensation. Izv AS USSR Ser Geogr 3:3-11

Burton AC, Edholm OG (1955) Man in a cold environment: physiological and pathological effects of exposure to low temperatures. Arnold,

Cadarette BS, Montain SJ, Kolka MA, Stroschein L, Matthew W, Sawka MN (1999) Cross validation of USARIEM heat strain prediction models Aviat Space Environ Med 70:996 - 1006

Chen L, Ng E (2013) Simulation of the effect of downtown greenery on thermal comfort in subtropical climate using PET index: a case study in Hong Kong Architectural Science Review 56:297-305
Dasler AR (1977) Heat stress, work function and physiological heat exposure limits in man. Washington, D.C, https://play.google.com/books/reader?id=49fL2qrLF8gC&printsec=frontcover&output=reader&authuser=0&hl=en&pg=GBS.PP2
Dayal D (1974) An Index for assessing heat stress in terms of physiological strain.
de Freitas C, Grigorieva E (2009) The Acclimatization Thermal Strain Index (ATSI): a preliminary study of the methodology applied to climatic conditions of the Russian Far East Int J Biometeorology 53:307 - 315
de Freitas C, Grigorieva E (2015) A comprehensive catalogue and classification of human thermal climate indices International Journal of Biometeorology 59:109–120 doi:10.1007/s00484-014-0819-3
de Freitas C, Grigorieva E (2016) A comparison and appraisal of a comprehensive range of human thermal climate indices Int J Biometeorology doi:10.1007/s00484-016-1228-6
de Freitas C, Ryken M (1989) Climate and physiological heat strain during exercise International Journal of Biometeorology 33:157-164 doi:10.1007/BF01084600
de Freitas CR (1986) Human thermal climates of New Zealand. New Zealand Meteorological Service, Misk Publ, 190, Wellington.
de Freitas CR (1987) Bioclimates Of Heat And Cold Stress In New Zealand Weather and Climate 7:55-60
de Freitas CR, Symon LV (1987) A Bioclimatic Index of Human Survival Times in the Antarctic Polar Record 23:651-659
de Paula Xavier AA, Lamberts R (2000) Indices of thermal comfort developed from field survey in Brazil ASHRAE Transactions 106:1-14
Dimiceli VE, Piltz SF, Amburn SA (2011) Estimation of Black Globe Temperature for Calculation of the Wet Bulb Globe Temperature Index Proceedings of the World Congress on Engineering and Computer Science 2
Dufton AF (1929) The eupatheostat Journal of Scientific Instruments 6:249
Eissing G (1995) Climate assessment indices Ergonomics 38:47–57 doi:10.1080/00140139508925084
Evans M (1980) Housing, climate, and comfort. Architectural Press, London
Falconer R (1968) Windchill, A Useful Wintertime Weather Variable Weatherwise 21:227 - 229, 255
Fanger PO (1970) Thermal Comfort - Analysis and Applications in Environmental Engineering. Danisch Technical Press, Copenhagen
Fanger PO, Melikov AK, Hanzawa H, Ring J (1988) Air Turbulence and Sensation of Draught Energy and Buildings 12:21 - 39
Frank A, Moran D, Epstein Y, Belokopytov M, Shapiro Y (1996) The estimation of heat tolerance by a new cumulative heat strain index. In: Shapiro Y, Moran D, Epstein Y (eds) Environmental Ergonomics: Recent Progress and New Frontiers. Freund Publishing House, Tel Aviv-London, pp 194 - 197
Gagge AP, Fobelets AP, Berglund LG (1986) A standard predictive index of human response to the thermal environment ASHRAE Transactions 92:709-731
Gagge AP, Nishi Y, Gonzalez RR (1973) Standard Effective Temperature - A single Temperature Index of Temperature Sensation and Thermal Discomfort. In: HMSO U (ed) Symposium on thermal comfort and moderate heat stress, Watford, UK, 1973 1973. Building Research Station,
Gagge AP, Stolwijk JAJ, Nishi Y (1971) An effective temperature scale based on a simple model of human physiological regulatory response ASHRAE TRANSACTIONS 77:21-36
Gallagher MJ, Robertson RJ, Goss FL, Nagle-Stilley EF, Schafer MA, Suyama J, Hostler D (2012) Development of a perceptual hyperthermia index to evaluate heat strain during treadmill exercise Europ J Appl Physiol 112:2025–2034
Givoni B (1976) Man, Climate and Architecture. Architectural Science Series, 2 edn. Applied Science Publ., Amsterdam
Givoni B, Goldman R (1972) Predicting rectal temperature response to work, environment, and clothing Journal of Applied Physiology 32:812-822
Givoni B, Goldman RF (1973) Predicting heart rate response to work, environment, and clothing ournal of Applied Physiology 34:201–204
Givoni B, Noguchi M, Saaroni H, Pochter O, Yaacov Y, Feller N, Becker S (2003) Outdoor comfort research issues Energy and Buildings 35:77-86 doi:10.1016/S0378-7788(02)00082-8
Goldberg V, Kurbjuhn C, Bernhofer C (2013) How relevant is urban planning for the thermal comfort of pedestrians? Numerical case studies in two districts of the City of Dresden (Saxony/Germany) Meteorologische Zeitschrift 22:739 - 751 doi:10.1127/0941-2948/2013/0463
Golovina EG, Rusanov VI (1993) Some questions in biometeorology. Russisches Staatshydrometeorologisches Institut, St Petersburg
Gonzalez RR, Berglund LG, Gagge AP (1978) Indices of thermoregulatory strain for moderate exercise in the heat J Appl Physiol Respir Environ Exerc Physiol 44:889-899

Gonzalez RR, Nishi Y, Gagge AP (1974) Experimental evaluation of standard effective temperature a new biometeorological index of man’s thermal discomfort International Journal Biometeorology 18:1-15 doi:10.1007/BF01450660
Graveling RA, Morris LA, Graves RJ (1988) Working in Hot Conditions in Mining: A Literature Review. Institute of Occupational Medicine Ergonomics Branch,
Gregorczuk M (1968) Bioclimates of the world related to air enthalpy International Journal of Biometeorology 12:35–39 doi:10.1007/BF01552976
Gregorczuk M, Cena K (1967) Distribution of Effective Temperature over the Surface of the Earth International Journal of Biometeorology 11:145-149
Hall J, Polte J (1960) Physiological index of strain and body heat storage in hyperthermia Journal of Applied Physiology 15:1027-1030
Hamdi M, Lachiver G, Michaud F (1999) A new predictive thermal sensation index of human response Energy and Buildings 29:167-178
He J (2011) A design supporting simulation system for predicting and evaluating the cool microclimate creating effect of passive evaporative cooling walls Building and Environment 46:584-596 doi:http://dx.doi.org/10.1016/j.buildenv.2010.09.005
He J, Hoyano A (2010) Measurement and evaluation of the summer microclimate in the semi-enclosed space under a membrane structure Building and Environment 45:230-242 doi:http://dx.doi.org/10.1016/j.buildenv.2009.06.006
Hedquist BC, Brazel AJ (2014) Seasonal variability of temperatures and outdoor human comfort in Phoenix, Arizona, U.S.A Building and Environment 72:377-388 doi:http://dx.doi.org/10.1016/j.buildenv.2013.11.018
Hill L, Hargood-Ash D (1919) On the cooling and evaporative powers of the atmosphere, as determined by the kata-thermometer Proceedings of the Royal Society of London B: Biological Sciences 90:438–447 doi:10.1098/rspb.1919.0004
Holmer I (1988) Assessment of cold stress in terms of required clothing insulation—ireq International Journal of Industrial Ergonomics 3:159-166 doi:10.1016/0169-8141(88)90017-0
Hori S (1978) Index for the assessment of heat tolerance J Human Ergol 7:135 - 144
Huang H, Ooka R, Kato S (2005) Urban thermal environment measurements and numerical simulation for an actual complex urban area covering a large district heating and cooling system in summer Atmospheric Environment 39:6362-6375 doi:http://dx.doi.org/10.1016/j.atmosenv.2005.07.018
Hubac M, Strelka F, Borsky I, Hubacova L (1989) Application of the relative summary climatic indices during work in heat for ergonomic purposes Ergonomics 32:733-750 doi:10.1080/00140138908966839
Jokl MV (1982) Standard Layers - A New Criterion of the Thermal Insulating Properties of Clothing Int J Biometeorology 26:37 - 48
Kakon AN, Mishima N, Kojima S (2009) Simulation of the urban thermal comfort in a high density tropical city: Analysis of the proposed urban construction rules for Dhaka, Bangladesh Building Simulation 2:291 doi:10.1007/s12273-009-9321-y
Kalkstein LS, Nichols MC (1996) A new spatial synoptic classification: Application to air-mass analysis International Journal Of Climatology 16:983 - 1004
Kalkstein LS, Valimont KM (1986) An evaluation of summer discomfort in the United States using a relative climatological index Bull Amer Meteor Soc 68:1535 - 1540
Kamon E, Ryan C (1981) Effective heat strain index using pocket computer American Industrial Hygiene Association Journal 42:611-615
Ketterer C, Ghasemi I, Bertram A, Kapp R (2013) Changes of thermal bioclimate through urban planning - Case study of Stuttgart-West Gefahrstoffe Reinhaltung der Luft 73:323 - 329
Ketterer C, Matzarakis A (2014) Human-biometeorological assessment of heat stress reduction by replanning measures in Stuttgart, Germany Landscape and Urban Planning 122:78-88 doi:http://dx.doi.org/10.1016/j.landurbplan.2013.11.003
Ketterer C, Matzarakis A (2015) Comparison of different methods for the assessment of the urban heat island in Stuttgart, Germany International Journal of Biometeorology 59:1299-1309 doi:10.1007/s00484-014-0940-3
Kobyscheva NB et al. (2008) Guidelines for specialized climatological services in economy. Ministry of Natural Resources and Ecology RussianFederation, Federal Service for Hydrometeorology and Environmental Monitoring, Saint Petersburg
Koenigsberger O, Mahoney C, Evans M (1971) Climate and House Design - Design of low-cost housing and cummunity facilities vol 1. United Nations Publication. United Nations, New York
Landsberg HE (1972) The assessment of human bioclimate. A limited review of physical parameters. Geneva, Switzerland
Lecha Estela LB (1998) Biometeorological classification of daily weather types for the humid tropics Int J Biometeorology 42:77 - 83

Lee DHK Proprioclimates of man and domestic animals. In: Climatology: reviews of research, UNESCO Conf. Paris, 1956, 1958.
Lee DHK, Henschel A (1966) Effects of physiological and clinical factors on response to heat Annals of the New York Academy of Sciences 134:743-749 doi:10.1111/j.1749-6632.1966.tb43059.x
Lee DHK, Vaughan JA (1964) Temperature Equivalent of Solar Radiation on Man Int J Biometeorology 8:61-69
Lind AR, Hellon RF (1957) Assessment of Physiological Severity of Hot Climates J Appl Physiol 11:35-40
Linke F (1926) Die Übertemperatur einer frei aufgestellten schwarzen Kugel Meteorologische Zeitschrift 42:11-15
Liopo TN, Cicenko GV (1971) Climatic conditions and human thermal state. Leningrad Hydrometeorological Publishing House.
Lobaccaro G, Acero JA (2015) Comparative analysis of green actions to improve outdoor thermal comfort inside typical urban street canyons Urban Climate 14, Part 2:251-267 doi:http://dx.doi.org/10.1016/j.uclim.2015.10.002
Lopes A, Correia E, Do Nascimento JM, Canario P (2014) Urban Bioclimate and Comfort Assessment in the African City of Praia (Cape Verde) Finisterra: Revista Portuguesa de Geografia 49:33-48
MacPherson RK (1962) The Assessment of the Thermal Environment. A Review British Journal of Industrial Medicine 19:151-164
Maloney SK, Forbes CF (2011) What effect will a few degrees of climate change have on human heat balance? Implications for human activity International Journal of Biometeorology 55:147-160 doi:10.1007/s00484-010-0320-6
Masterson J, Richardson FA (1979) Humidex, a method of quantifying human discomfort due to excessive heat and humidity. Environment Canada.
Matyukhin VA, Kushnirenko EY (1987) Complex quality assessment of environmental influence on the human body. In: Proceedings of the WMO; WHO, UNEP-Symposium on Climate and Human Health, Leningrad, 1986. WMO-WCP, Geneva, pp 33-37
McArdle B, Dunham W, Holling HE, Ladell WSS, Scott JW, Thomson ML, Weiner JS (1947) The Prediciton of the physiological Effects of warm and hot environments vol RNP Rep. 47/391.
McIntyre D (1973) A guide to thermal comfort Applied Ergonomics 4:66-72 doi:10.1016/0003-6870(73)90079-3
McLaughlin JT, Shulman MD (1977) An Anthropocentric Summer Severity Index Int J Biometeorology 21:16 - 28
McPherson MJ The generalization of air cooling power. In: Africa MVSoS (ed) Proceedings of the 5th International Mine Ventilation Congress, 1992. Johannesburg:, pp 27 - 35
Mehnert P, Malchaire J, Kampmann B, Piette A, Griefahn B, Gebhardt H (2000) Prediction of the average skin temperature in warm and hot environments Eur J Appl Physiol 82:52-60
Minella FO, Krüger E, Honjo S, Goyette S, Hedjazi A (2014) Daytime microclimatic impacts of the SOVALP project in summer: A case study in Geneva, Switzerland Simulation 90:857-873 doi:10.1177/0037549714543085
Mochida T (1979) Comfort Chart : An Index for Evaluating Thermal Sensation Memoirs of the Faculty of Engineering, Hokkaido University 15:175 - 185
Moher D, Liberati A, Tetzlaff J, Altman DG, The PG (2009) Preferred Reporting Items for Systematic Reviews and Meta-Analyses: The PRISMA Statement PLOS Medicine 6:e1000097 doi:10.1371/journal.pmed.1000097
Morakinyo TE, Dahanayake KWDKC, Adegun OB, Balogun AA (2016) Modelling the effect of tree-shading on summer indoor and outdoor thermal condition of two similar buildings in a Nigerian university Energy and Buildings 130:721-732 doi:http://dx.doi.org/10.1016/j.enbuild.2016.08.087
Morakinyo TE, Lam YF (2016) Simulation study on the impact of tree-configuration, planting pattern and wind condition on street-canyon's micro-climate and thermal comfort Building and Environment 103:262-275 doi:http://dx.doi.org/10.1016/j.buildenv.2016.04.025
Moran DS, Castellani JW, O'Brien C, Young DT, Pandolf KB (1999) Evaluating physiological strain during cold exposure using a new cold strain index Am J Physiol 277:R556–R564
Moran DS, Pandolf KB, Shapiro Y, Heled Y, Shani Y, Mathew WT, Gonzalez RR (2001) An environmental stress index (ESI) as a substitute for the wet bulb globe temperature (WBGT) Journal of Thermal Biology 26:427 - 431
Moran DS, Shitzer A, Pandolf KB (1998) A physiological strain index to evaluate heat stress Am J Physiol Regul Integr Comp Physiol 275
Mount L, Brown D (1985) The calculation from weather records of the requirement for clothing insulation International Journal of Biometeorology 29:311-321 doi:10.1007/BF02189205
Müller N, Kuttler W, Barlag A-B (2014) Counteracting urban climate change: adaptation measures and their effect on thermal comfort Theoretical and Applied Climatology 115:243-257 doi:10.1007/s00704-013-0890-4

Office of the Federal Coordinator for Meteorological services and supporting research (2003) Report on Wind Chill Temperature and Extreme Heat Indices: Evaluation and Improvement Projects. Washington, DC
Ono H, Kawamura T (1991) Sensible climates in monsoon Asia International Journal of Biometeorology 35:39-47 doi:10.1007/BF01040962
Osczevski R, Bluestein M (2005) The New Wind Chill Equivalent Temperature Chart Bull Amer Meteor Soc 86:1453-1458
Pandolf KB, Stroschein LA, Drolet LL, Gonzalez RR, Sawka MN (1985) Prediction Modeling of Physiological Responses and Human Performance in the Heat. US Army Rsch Inst of Env Med, Natick, Massachusetts
Park S, Tuller S, Jo M (2014) Application of Universal Thermal Climate Index (UTCI) for microclimatic analysis in urban thermal environments Landscape and Urban Planning 125:146 - 155 doi:http://dx.doi.org/10.1016/j.landurbplan.2014.02.014
Parsons KC (2014) Human thermal environments: the effects of hot, moderate, and cold environments on human health, comfort, and performance. 3 edn. CRC Press,
Peng LL, Jim YC (2013) Green-Roof Effects on Neighborhood Microclimate and Human Thermal Sensation Energies 6 doi:10.3390/en6020598
Pepi JW (1987) The summer simmer index Weatherwise 40:143-145
Pepi JW (1999) The new Summer Simmer Index: a comfort index for the new millennium, http://www.summersimmer.com/home.htm.
Pulket C, Henschel A, Burg WR, Saltzman BE (1980) A comparison of heat stress indices in a hot-humid environment American Industrial Hygiene Association Journal 41:442-449
Qaid A, Bin Lamit H, Ossen DR, Raja Shahminan RN (2016) Urban heat island and thermal comfort conditions at micro-climate scale in a tropical planned city Energy and Buildings 133:577-595 doi:http://dx.doi.org/10.1016/j.enbuild.2016.10.006
Robinson S, Turrell ES, Gerking SD (1944) Physiologically equivalent conditions of air temperature and humidity Am J Physiol 143:21-32
Robitu M, Musy M, Inard C, Groleau D (2006) Modeling the influence of vegetation and water pond on urban microclimate Solar Energy 80:435-447 doi:http://dx.doi.org/10.1016/j.solener.2005.06.015
Rodriguez C, Mateos J, Garmendia J (1985) Biometeorological comfort index International Journal of Biometeorology 29:121-129 doi:10.1007/BF02189031
Rohles FH, Hayter R, Milliken G (1975) Effective Temperature (ET*) as a predictor of thermal comfort ASHRAE Trans 81:148-156
Rohles FH, Nevis RG (1971) The nature of thermal comfort for sedentary man ASHRAE Trans 77:239-246
Rohles FH, Woods JE, Nevis RG The Effects of Air Movement and Temperature on the Thermal Sensation of Sedentary Man. In: ASHRAE Semiannual Meeting, Los Angeles CA, 1974.
Rublack K, Medvedeva EF, Gaebelein H, Noack H, Schulz G (1981) Integrative Bewertung der Wärmebelastung durch Arbeit und Klima Z ges Hyg 27:12 - 17
Rusanov V (1981) Complex meteorological indices and methods of climate assessment in medical purposes. Tomsk Tomsk State University
Rusanov V Climate and human health. In: WMO; WHO, UNEP-Symposium on Climate and Human Health in, Leningrad 1986 1987. WMO-WCP, Geneva, pp 101–106
Santee WR, Wallace RF (2003) Evaluation of weather service heat indices using the USARIEM heat strain decision aid (HSDA) model. U.S. Army Research Institute of Environmental Medicine, Natick, MA
Scharlau K (1943) Die Schwüle als meßbare Größe Bioklimatische Beiblätter (Meteorologische Zeitschrift) 10:19-23
Schoen C (2005) A New Empirical Model of the Temperature–Humidity Index J Appl Meteor 44
Schrijvers PJC, Jonker HJJ, de Roode SR, Kenjereš S (2016) The effect of using a high-albedo material on the Universal Temperature Climate Index within a street canyon Urban Climate 17:284-303 doi:http://dx.doi.org/10.1016/j.uclim.2016.02.005
Sheridan SC (2002) The redevelopment of a weather-type classification scheme for North America International Journal Of Climatology 22:51-68 doi:10.1002/joc.709
Siple PA, Passel CF (1945) Measurements of Dry Atmospheric Cooling in Subfreezing Temperatures Proceedings of the American Philosophical Society 89:177-199
Smith FE (1952) Effective Temeprature as an index of physiological stress. Medical Research Council, London
Stavrakakis GM, Tzanaki E, Genetzaki VI, Anagnostakis G, Galetakis G, Grigorakis E (2012) A computational methodology for effective bioclimatic-design applications in the urban environment Sustainable Cities and Society 4:41-57 doi:http://dx.doi.org/10.1016/j.scs.2012.05.002
Steadman RG (1971) Indices of Windchill of Clothed Persons J Appl Meteor 10:674-683
Steadman RG (1979) The Assessment of Sultriness. Part II: Effects of Wind, Extra Radiation and Barometric Pressure on Apparent Temperature Journal of Applied Meteorology 18:874 - 885
Steadman RG (1984) A Universal Scale of Apparent Temperature J Climate Appl Meteor 23:1674-1687

Steadman RG (1994) Norms of apparent temperature in Australia Aust Met Mag 43:1-16
Stull R (2011) Wet-Bulb Temperature from Relative Humidity and Air Temperature Journal of Applied Meteorology and Climatology 50:2267-2269 doi:10.1175/JAMC-D-11-0143.1
Taleghani M, Kleerekoper L, Tenpierik M, van den Dobbelsteen A (2015) Outdoor thermal comfort within five different urban forms in the Netherlands Building and Environment 83:65-78 doi:http://dx.doi.org/10.1016/j.buildenv.2014.03.014
Taleghani M, Sailor D, Ban-Weiss GA (2016) Micrometeorological simulations to predict the impacts of heat mitigation strategies on pedestrian thermal comfort in a Los Angeles neighborhood Environmental Research Letters 11:024003
Tennenbaum J, Sohar E, Adar R, Gilat T, Yaski D (1961) The physiological significance of the cumulative discomfort index (Cum DI) Harefuah 60:315-319
Terjung W (1966) Physiologic climates of the conterminous united states: A bioclimatic classification based on man Annals of the Association of American Geographers 56:141-179 doi:10.1111/j.1467-8306.1966.tb00549.x
Terjung W (1968) World patterns of distribution of the monthly comfort index Int J Biometeorology 12:119-151
Thilenius R, Dorno C (1925) Das Davoser Frigorimeter - ein Instrument zur Dauerregistrierung der physiologischen Abkühlungsgröße Meteorologische Zeitschrift 42:57-60
Tikuisis P, Mclellan TM, Selkirk G (2002) Perceptual versus physiological heat strain during exercise-heat stress Med Sci Sports Exerc 34:1454 - 1461
Tromp SW (1966) A Physiological Method for Determining the Degree of Meteorological Cooling Nature 210:486-487
Tumini I, Higueras García E, Baereswyl Rada S (2016) Urban microclimate and thermal comfort modelling: strategies for urban renovation International Journal of Sustainable Building Technology and Urban Development 7:22-37 doi:10.1080/2093761x.2016.1152204
Tzenkova A, Ivancheva J, Koleva E, Videnov P (2007) The human comfort conditions at Bulgarian Black Sea side. Paper presented at the Developments in Tourism Climatology, 3rd International Workshop on Climate, Tourism and Recreation, Alexandroupolis, Greece, December 2007
Vernon HM, Warner CG (1932) The influence of the humidity of the air on capacity for work at high temperatures J Hyg 32:431 - 462
Vogt JJ, Candas V, Libert JP (1982) Graphical determination of heat tolerance limits Ergonomics 25:285-294 doi:10.1080/00140138208924955
Vogt JJ, Candas V, Libert JP, Daull F (1981) Required Sweat Rate as an Index of Thermal Strain in Industry Original vol 10. Elsevier Scientific Publishing Company,
Wallace RF, Kriebel D, Punnett L, Wegman DH, Wenger CB, Gardner JW, Gonzalez RR (2005) The effects of continuous hot weather training on risk of exertional heat illness Med Sci Sports Exerc 37:84-90
Watts JD, Kalkstein LS (2004) The Development of a Warm-Weather Relative Stress Index for Environmental Applications Journal of Applied Meteorology 43:503 - 513
Webb GG (1959) An Analysis Of Some Observations Of Thermal Comfort In An Equatorial Climate Brit J industr Med 16:297-310
Weiss M (1982) The humisery and other measures of summer discomfort Nat Weather Digest 7:10-18
Wenzel HG (1978) Heat stress upon undressed man due to different combinations of elevated environmental temperature, air humidity, and metabolic heat production: a critical comparison of heat stress indices J Hum Ergol 7:185–206
Winslow CEA, Gagge AP, Greenburg L, Moriyama IM, Rodee EJ (1935) The calibration of the thermo-integrator Am J Hygiene 22:137-156
Winslow CEA, Herrington LP (1949) Temperature and Human Life. Princeton University Press, Princeton
Winslow CEA, Herrington LP, Gagge AP (1937) Physiological reactions of the human body to varying environmental temperatures Am J Physiol 120:1-22
Yaglou CP, Minard D (1957) Control of heat casualties atmilitary training centers Arch Indust Health 16:302 - 316
Yahia MW, Johansson E (2014) Landscape interventions in improving thermal comfort in the hot dry city of Damascus, Syria—The example of residential spaces with detached buildings Landscape and Urban Planning 125:1-16 doi:http://dx.doi.org/10.1016/j.landurbplan.2014.01.014
Yang F, Lau SSY, Qian F (2011) Thermal comfort effects of urban design strategies in high-rise urban environments in a sub-tropical climate Architectural Science Review 54:285-304 doi:10.1080/00038628.2011.613646
Yang W, Wong NH, Lin Y (2015) Thermal Comfort in High-rise Urban Environments in Singapore Procedia Engineering 121:2125-2131 doi:http://dx.doi.org/10.1016/j.proeng.2015.09.083
Young KC (1979) The influence of environmental parameters on heat stress during exercise Journal of Applied Meteorology 18:886-897

Zaninovic K (1992) Limits of warm and cold bioclimatic stress in different climatic regions Theoretical and Applied Climatology 45:65-70 doi:10.1007/BF00865996

Zhang W, Mak CM, Ai ZT, Siu WM (2012) A Study of the Ventilation and Thermal Comfort of the Environment Surrounding a New University Building under Construction Indoor and Built Environment 21:568-582 doi:10.1177/1420326x11419871

Zölch T, Maderspacher J, Wamsler C, Pauleit S (2016) Using green infrastructure for urban climate-proofing: An evaluation of heat mitigation measures at the micro-scale Urban Forestry & Urban Greening 20:305-316 doi:http://dx.doi.org/10.1016/j.ufug.2016.09.011
